# Aggregation-induced emission: mechanistic study of the clusteroluminescence of tetrathienylethene†
†Electronic supplementary information (ESI) available: The supplementary information is reported: the crystal data of TTE, sl-TTE and DTE, the photophysical data of DTE, and the optical data and UV-vis spectra of TTE, sl-TTE and fl-TTE. The crystallographic data for TTE (1509230), DTE (1509231), and sl-TTE (1509232) have been submitted to the Cambridge Crystal Data Centre (CCDC). For ESI and crystallographic data in CIF or other electronic format see DOI: 10.1039/c6sc05192h



**DOI:** 10.1039/c6sc05192h

**Published:** 2017-01-11

**Authors:** Lucia Viglianti, Nelson L. C. Leung, Ni Xie, Xinggui Gu, Herman H. Y. Sung, Qian Miao, Ian D. Williams, Emanuela Licandro, Ben Zhong Tang

**Affiliations:** a HKUST Shenzhen Research Institute , No. 9 Yuexing 1st Road, South Area, Hi-tech Park, Nanshan , Shenzhen 518057 , China; b Department of Chemistry , Hong Kong Branch of Chinese National Engineering Research Center for Tissue Restoration and Reconstruction , Institute for Advanced Study , Division of Biomedical Engineering , Division of Life Science , State Key Laboratory of Molecular Neuroscience , Institute of Molecular Functional Materials , The Hong Kong University of Science and Technology , Clear Water Bay , Kowloon , Hong Kong , China . Email: tangbenz@ust.hk; c Guangdong Innovative Research Team , SCUT-HKUST Joint Research Laboratory , State Key Laboratory of Luminescent Materials and Devices , South China University of Technology , Guangzhou 510640 , China; d Department of Chemistry , The Chinese University of Hong Kong , Shatin, New Territories , Hong Kong , China; e Dipartimento di Chimica , Università degli Studi di Milano , Via Golgi 19 , 20133 , Milano , Italy

## Abstract

In this work we have investigated the aggregation-induced emission (AIE) behaviour of 1,1,2,2-tetra(thiophen-2-yl)ethene (tetrathienylethene, TTE). The semi-locked and fully-locked derivatives (sl-TTE and fl-TTE) have been synthesized to better understand the mechanism behind the solid state photoluminescence of TTE. TTE is a typical AIEgen and its luminescence can be explained through the mechanistic understanding of the restriction of intramolecular motions (RIM). The emissive behaviour of TTE in the THF/water aggregates and crystal state have also been studied, revealing a remarkable red-shift of 35 nm. A similar red-shift emission of 37 nm from the THF/water aggregates to the crystal state is also observed for (*E*)-1,2-di(thiophen-2-yl)ethene (*trans*-dithienylethene, DTE). Crystal analysis has revealed that the emission red-shifts are ascribable to the presence of strong sulfur–sulfur (S···S) intra- and intermolecular interactions that are as close as 3.669 Å for TTE and 3.679 Å for DTE. These heteroatom interactions could help explain the photoluminescence of non-conventional luminophores as well as the luminescence of non-conjugated biomacromolecules.

## Abstract 2

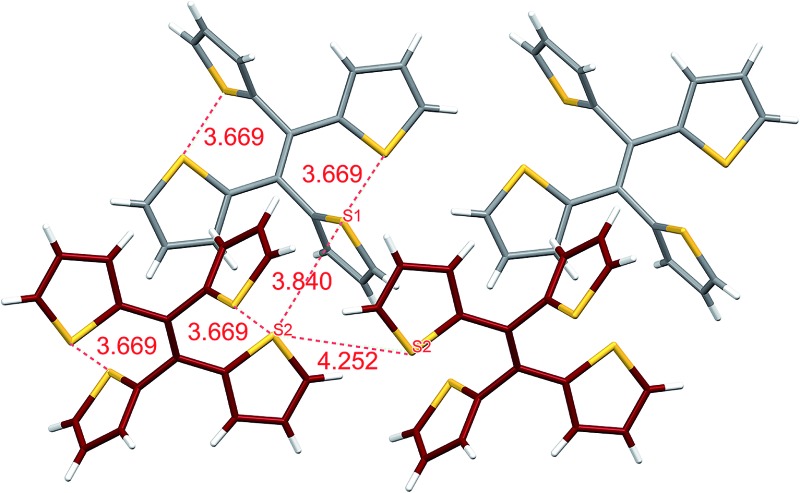
Crystallization induced S···S interactions leading to an unusual luminescent phenomenon.

## Introduction

Luminescence has continued to light the way in many important research fronts. In recent years, researchers worldwide have continually developed and improved luminescent materials in the fields of OLEDs,^1–3^ light emitting organic field effect transistors (LEOFETs),^4–6^ bioimaging agents,^7,8^ and super resolution microscopy.^9–11^ Many of these scaffolds are often designed with rigid, planar structures as this enhances conjugation, allowing researchers to tune and produce the desired emission wavelength. However, many of these planar systems undergo a phenomenon known as aggregation-caused quenching (ACQ). Intermolecular interactions that quench luminescence occur when the molecules are in close proximity to each other, enhancing non-radiative decay pathways of the molecules and resulting in a loss of emission. For systems where the emission intensity is highly affected by concentration effects, a delicate balance is required to find the optimal working conditions, as both high and low concentration results in a weak intensity due to quenching or a lack of dye molecules, respectively. The next generation of luminescent materials will need to overcome this ACQ phenomenon while maintaining desirable properties such as strong emission intensity. One method to guide the production of the next generation of luminogens is to study and understand existing systems that are unaffected by the ACQ phenomenon. Drawing a mechanistic understanding from these molecules could allow for those principles to be applied in the rational synthesis of new systems.

One such research development in the field of luminescence is the aggregation-induced emission (AIE) platform.^12^ AIE research first started in order to understand poor solution state but strong solid state emission from the structural derivative of hexaphenylsilole (HPS), an archetypal AIEgen.^13^ The mechanistic understanding of the AIE process has currently shown that molecular motions such as vibrations or rotations, *e.g.* phenyl ring rotations in HPS, deactivate radiative pathways in the solution state, rendering the molecule virtually non-emissive. In contrast, the restriction of intramolecular motions (RIM) taking place in the aggregate or solid state due to physical constraints blocks non-radiative decay and restores emission. Capitalizing on this understanding has led to a boom in the development of a large array of AIEgens that show exceptional performance in many areas such as OLEDs,^14–18^ bioimaging,^19–23^ chemosensing,^24–28^ and stimuli responsive materials.^29–34^

In the continual process of understanding luminescence and developing new emissive scaffolds, researchers have increasingly reported on new AIE systems that cannot be adequately explained using the current understanding of photophysics. Displaying strong emission in the aggregated or solid state, many of these systems lack conventional chromophores, *i.e.* conjugated π-systems, in their molecular structures. For instance, something that is particularly hard to explain is the luminescence observed in poly(isobutene succinic anhydride),^35^ poly(maleic anhydride) (PMAh),^36^ polyurea,^37^ and even starch.^38^ All of these systems lack any sort of conjugated units but produce blue emission in the aggregate or solid state. One common attribute that many of these non-conventional luminescent systems share is electron-rich heteroatoms. It has been observed that these heteroatoms are often in close proximity to each other, especially when the polymers adopt a folded conformation. The clustering of heteroatoms can result in observable emission. Thus, the displayed luminescence can be defined as “clusteroluminescence”.

Could through-space interactions between heteroatoms be key to the observed luminescence? We have proposed the theory of clusteroluminescence^12^ to explain the luminescent phenomena of these non-conventional systems; close through-space interactions between the heteroatoms could create a new channel to radiatively dissipate energy, which turns on luminescence. The clusteroluminogen hypothesis mirrors the mechanism of the AIE molecules. When the molecules are well dissolved, the excitation energy is easily dissipated *via* molecular motions. Upon aggregation, visible emission is observed if two important criteria are met: (1) the molecular motions that dissipated the energy become blocked, and more importantly (2) the heteroatoms come into close proximity of each other such that close through-space interactions can take place. Understanding the cause of this photophysical phenomenon could have far reaching implications. For instance, biomolecules often have a rich array of heteroatoms incorporated into their structure. Grasping the mechanics of these systems could allow us to create new luminogens with enhanced properties. This can also help us to identify new sources of autofluorescence in bio-systems, which might allow us to overcome this obstacle in the future.

There has been an increasing number of published studies regarding weak through-space nonbonding interactions between second and third row heteroatoms.^39–43^ These interactions have shown a gain in the stabilization of the system. Stabilizing nonbonding through-space interactions has even been reported in the 1960s in dioxane species.^44,45^ Indeed, it has been described that these nonbonding interactions can form a new set of orbitals that could narrow the energy gap between the HOMO and LUMO of the system.^46,47^ These reports corroborate the clusteroluminogen hypothesis. Unfortunately, many of the reported luminescent systems that lack conjugated π-networks and are rich in heteroatoms are polymeric in nature, and so renders a detailed study into the heteroatom interactions nearly impossible.

**Chart 1:**
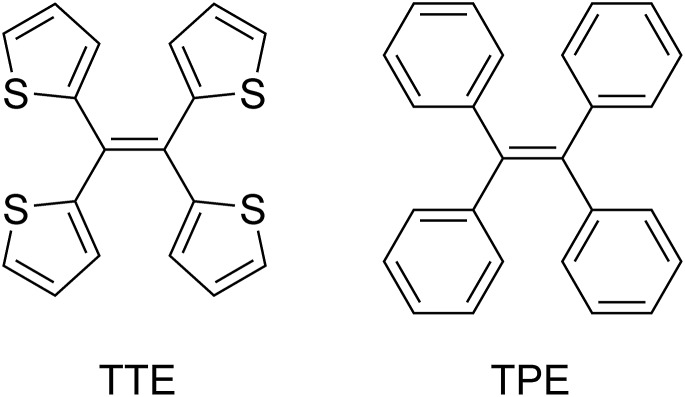
Chemical structure of tetrathienylethene (TTE) and tetraphenylethene (TPE).

**Scheme 1:**
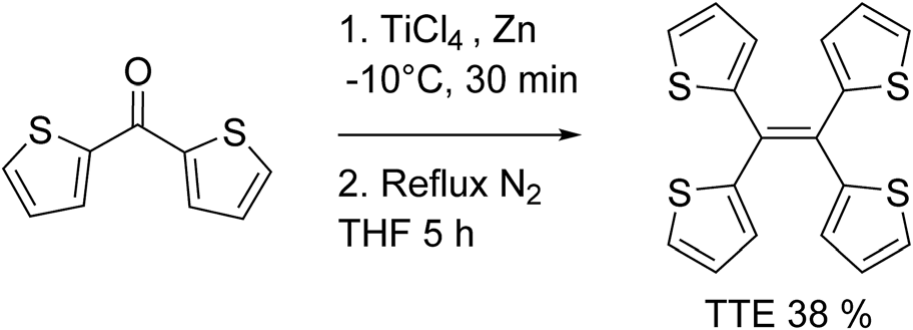
Synthesis of tetrathienylethene (TTE) *via* McMurry coupling.

**Scheme 2:**
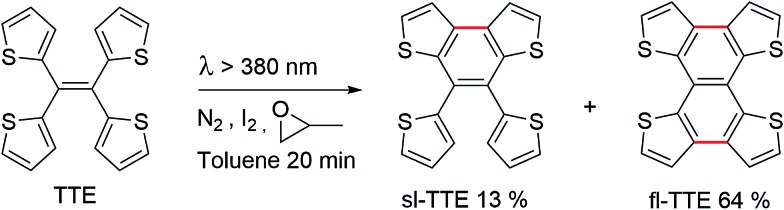
Synthesis of semi-locked TTE (sl-TTE) and fully-locked TTE (fl-TTE).

If there was a small molecular system that also exhibited strong heteroatom interactions, we could use it as a model to explain the clusteroluminescence of non-conventional chromophores. Herein, we report a small molecule, 1,1,2,2-tetra(thiophene-2-yl)ethene (tetrathienylethene, TTE, Chart 1 and Scheme 1), whose AIE feature was qualitatively observed previously.^48^ In this work we have thoroughly investigated its AIE behaviour, and have rationalized the mechanism behind it, by synthesizing its locked counterpart, tetrathieno[2,3-*a*:3′,2′-*c*:2′′,3′′-*f*:3′′′,2′′′-*h*]-naphthalene^49^ (fully-locked TTE, fl-TTE, Scheme 2). The AIE mechanism of TTE accounts for the restriction of the intramolecular rotations (RIR). Similar to other AIE luminogens (AIEgens), TTE also exhibits the morphochromism phenomenon, since there is a change in the emission going from the THF/water aggregate to the crystal. However, its morphochromism is unusual with respect to other AIEgens, since a red-shift (rather than a blue-shift) of 35 nm occurs from its emission in the THF/water aggregates to its crystal emission. Crystal analysis showed that due to the spatial arrangement of the thiophene rings in the crystal, no π–π interactions exist, while strong intra- and intermolecular S···S interactions have been found, with a measured distance as close as 3.669 Å, which is below the theoretical equilibrium value of 4.0 Å.^50^ By investigating the photoluminescence (PL) behaviour of the lower homolog of the thiophene-substituted ethene series, (*E*)-1,2-di(thiophene-2-yl)ethene (*trans*-dithienylethene, DTE), we have encountered a similar phenomenon; a 36 nm red-shift emission occurs when comparing the THF/water aggregate emission from DTE to its crystal emission. By analysing the crystal structure of DTE, we have found a similar S···S interaction with a distance of 3.679 Å. As no π–π interactions were found in either TTE or DTE, the red-shifts in emission in both of these systems seem to be a result of the through-space S···S interactions. Detailed studies of the photophysical properties and crystal structure analysis of TTE are presented below. These are compared and contrasted with the archetypal AIEgen tetraphenylethene (TPE, Chart 1) and the other synthesized TTE derivatives to better understand the photophysics behind the AIE mechanism and the through-space interactions.

## Results and discussion

### Photophysical properties

TTE has been synthesized *via* McMurry coupling as shown in Scheme 1.^51^

**Fig. 1:**
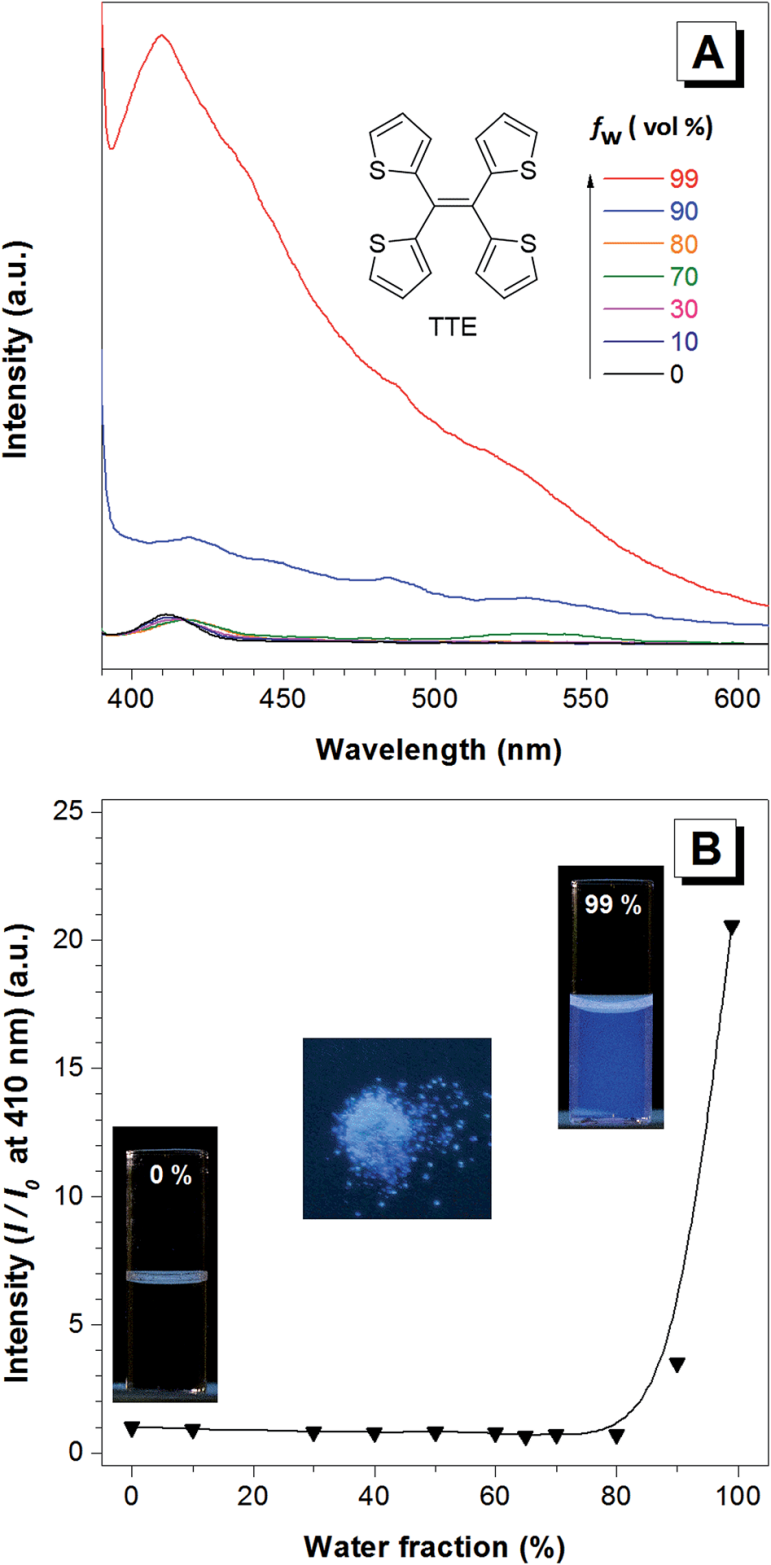
The AIE features of the TTE molecule. (A) The photoluminescence (PL) spectra of TTE in THF and THF/water mixtures with increasing water fractions (*f*_w_) to 99%. (B) Change in the PL intensity of TTE at 410 nm *versus* the water fraction in the THF/water mixtures. Concentration: 10^–5^ M. Excitation at 368 nm. Inset (from left to right): fluorescence of TTE in pure THF, powder, and in THF/water mixture at 99% *f*_w_.

Having a similar structure to the TPE, TTE also has good solubility in common organic solvents such as THF, dichloromethane, and chloroform, and due to its hydrophobic nature, it has poor solubility in water. Therefore, we chose a THF/water mixture system to investigate its luminescent behaviour upon aggregation. The pure THF solution of TTE is virtually non-emissive (Fig. 1A and B, inset).

When the fraction of the poor solvent, water, is increased while keeping a fixed TTE concentration of 10^–5^ M, the emission intensity increases with the maximum peak observed at 410 nm. Fig. 1B shows the emission intensity enhancement with increasing water fraction. Faint emission is detectable in the pure THF solution (0% water fraction), but no prominent enhancement is observed until the water fraction reaches 90%. The proposed RIM mechanism^12^ is employed to explain the above data. In the solution state, the TTE molecules experience minimal interactions between each other. Surrounded by solvent molecules, this allows each of the thiophene rings of TTE to freely rotate and vibrate. Upon excitation, this freedom causes the molecules to relax back to the ground state *via* non-radiative processes, resulting in weak solution emission. With increasing water volume fractions, the hydrophobic molecules aggregate together. The proximity of the neighbouring molecules induces steric effects which block the intramolecular motions of TTE thus preventing relaxation *via* non-radiative channels. With the excited state non-radiative decay processes prohibited upon aggregation, the emission becomes strongly enhanced.^52^ It is worth noting that TTE molecules only begin to show emission enhancement at a high water fraction of 90%, whereas TPE exhibits emission enhancement when the water fraction is above 60%.^53^

**Fig. 2:**
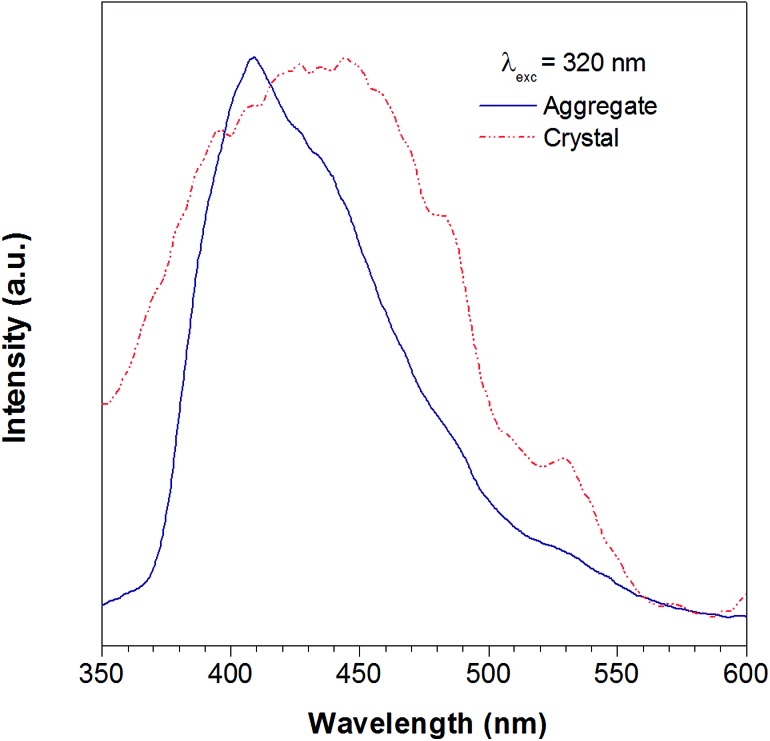
The photoluminescence spectra of the TTE THF/water aggregates (blue line) and crystal (red line). Excitation at 320 nm.

According to the reported method as described in the experimental section, we successfully obtained the crystal of TTE. To further study the luminescent properties of solid state TTE, we have conducted a photoluminescence study of TTE in the crystal and THF/water aggregation states (Fig. 2). These photoluminescence studies were performed using an excitation wavelength of 320 nm. The emission maximum peak for the crystal was measured at 444 nm and at 410 nm for the THF/water aggregate. Further discussion and explanation of the photophysical properties of TTE will be included in the following crystal packing section.

Currently, restriction of intramolecular motion (RIM) is one of the leading theories for explaining AIE phenomena.^12,54^ To explore the AIE activity in the TTE system, we adopted a feasible synthetic route to obtain the locked structures of TTE (Scheme 2),^49^ 4,5-bis(thiophene-2-yl)thieno[3,2-*e*]benzo[*b*]thiophene, (semi-locked TTE, sl-TTE) and tetrathieno[2,3-*a*:3′,2′-*c*:2′′,3′′-*f*:3′′′,2′′′-*h*]-naphthalene (fully-locked TTE, fl-TTE). The sl-TTE compound was also further characterized using single crystal X-ray diffraction (Fig. SI13–19†).

**Fig. 3:**
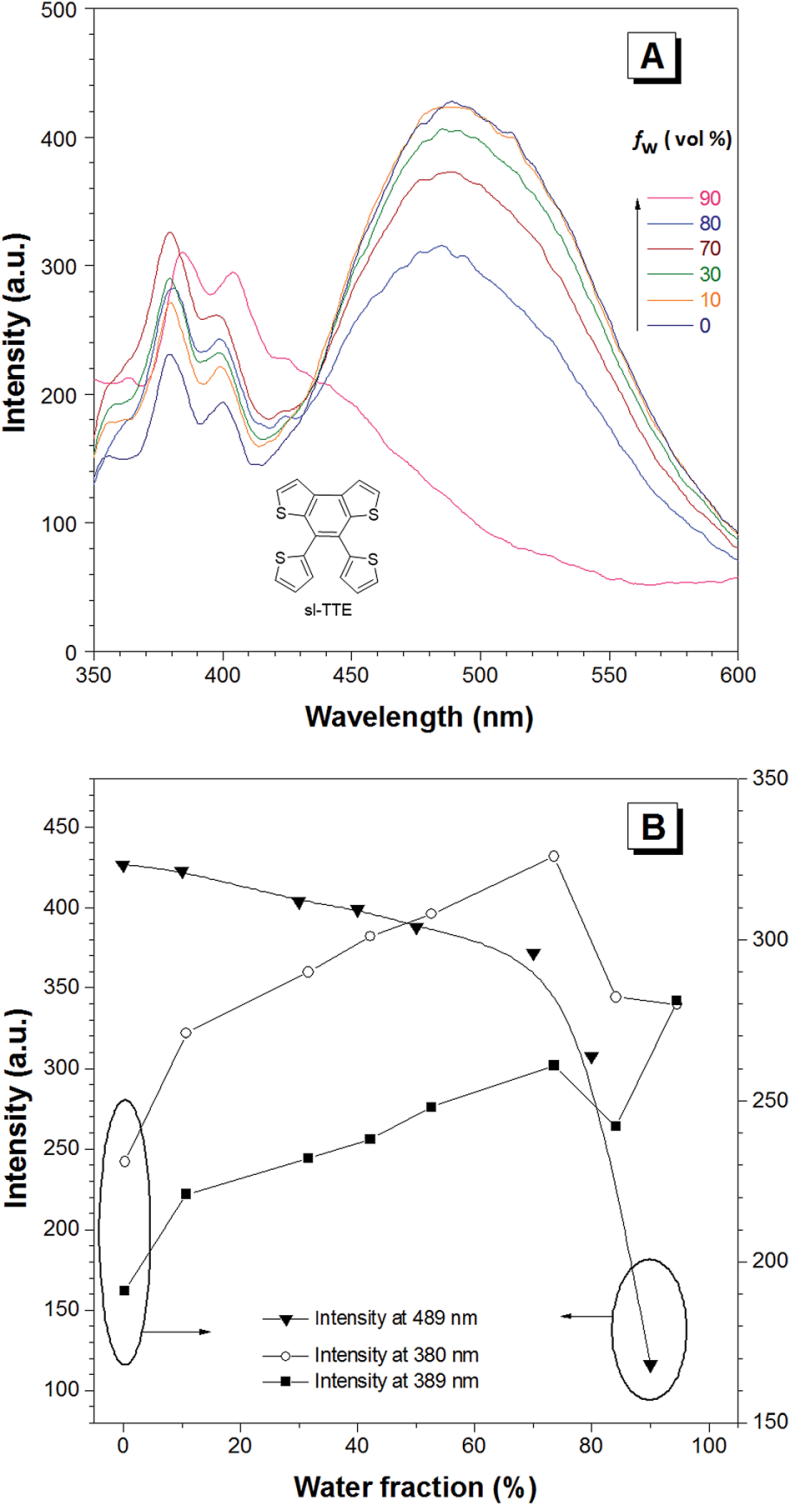
The AIE and ACQ features of the sl-TTE molecule. (A) The photoluminescence (PL) spectra of sl-TTE in THF and THF/water mixtures with increasing water fractions (*f*_w_) up to 90%. (B) Change in the PL intensity of sl-TTE at: 489, 389 and 380 nm *versus* the water fraction in the THF/water mixtures. Concentration: 10^–5^ M. Excitation at 323 nm.

**Fig. 4:**
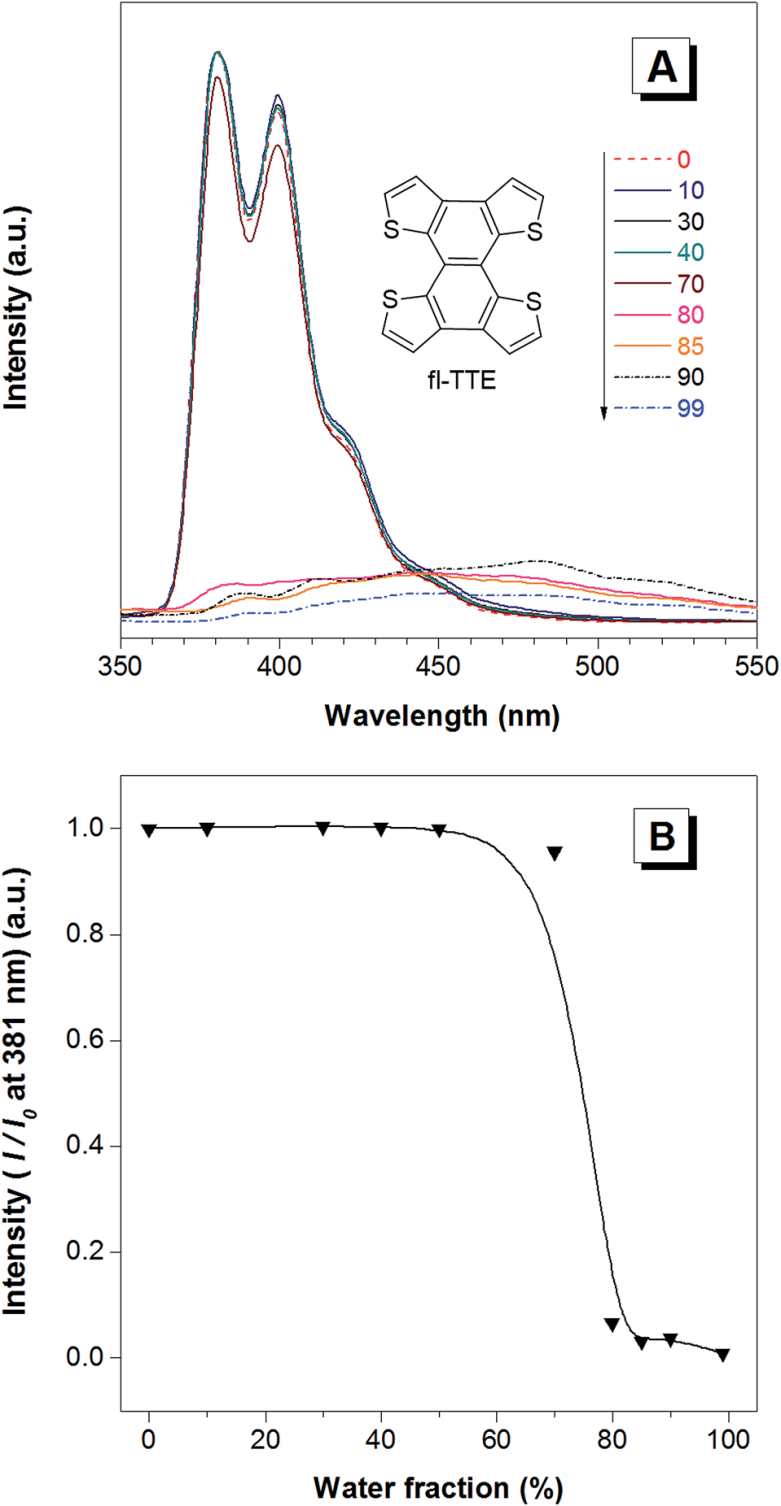
The ACQ features of the fl-TTE molecule. (A) The photoluminescence (PL) spectra of fl-TTE in THF and THF/water mixtures with increasing water fractions (*f*_w_) up to 90%. (B) Change in the PL intensity of fl-TTE at 381 nm *versus* the water fraction in the THF/water mixtures. Concentration: 10^–5^ M. Excitation at 299 nm.

The photoluminescence experiments of sl-TTE and fl-TTE were conducted and the data is shown in Fig. 3 and 4, respectively. Using the same experimental conditions that were used for TTE, sl-TTE was also studied with the THF/water mixture system. Starting from a pure THF solution, which has 0% water fraction, two out of the three fluorescent peaks are detected in the ultraviolet region while the third one is located at 489 nm (Fig. 3A). Upon increasing the water fraction, both ultraviolet peaks are enhanced in terms of the emission intensity. In contrast, the fluorescence emission at 489 nm is dramatically decreased. Comparing the 0% and 90% water fractions, the fluorescence at 489 nm experiences a 0.25 fold change while the fluorescent peaks below 400 nm increased with a 1.47 fold change as shown in Fig. 3B. The broad unstructured band at 489 nm is likely due to emission from the benzodithiophene core conjugated with the thiophene rings. The rotations of the rings change the conjugation efficiency between the core and the thienyl rings, resulting in the broad and unstructured nature of the emission band. As the molecules aggregate, the emission intensity of this broad band is reduced, indicating that the original electronic conjugation is disrupted. This electronic disruption could be due to the formation of intermolecular steric interactions that twist the thienyl rings out-of-plane with the benzodithiophene core. These intermolecular steric interactions also rigidify the molecules, resulting in the increase in the emission intensity of the peaks below 400 nm. The peaks below 400 nm are attributable to emission of the thiophene rings. Indeed, the two freely rotatable thiophenes, like AIEgens, decrease radiative decay in the solution by dissipating the energy through vibrational motions. However, in the aggregation state, the thiophene rings become more emissive as a result of restriction of intramolecular motions of sl-TTE, specifically the rotational motion of the thiophene rings and vibrational motion of the whole molecule. This causes emission in the ultraviolet region to be stronger in the aggregation state than in the solution state. In this way, sl-TTE exhibits both ACQ and AIE characteristics.

The fully locked structure of TTE (fl-TTE) displays photoluminescent properties that are much more similar to that of conventional luminophores. In Fig. 4A, the photoluminescence spectrum is recorded with the same methodology used for the previous compounds. Strong emission is observed in the solution state. When the water fraction of the solution is increased, the emission decreases and the maximum emission peak is red-shifted. Due to the fully locked structure, its discotic shape endows the molecule with the ACQ effect, as shown in Fig. 4B. Potential intensive π–π stacking interactions between the molecules can lead to a narrowing of the energy gap of the molecule upon aggregation. As there are no freely rotatable moieties in fl-TTE, no AIE activity is observed for this molecule. As is typical of ACQ dyes, fl-TTE is emissive in the solution state with two peaks as a result of its rigid nature. When the water fraction reaches 90%, the emission is greatly weakened which is likely to be due to the formation of excimers, as evidenced by the red-shift in the emission to the visible region of the spectrum.

**Fig. 5:**
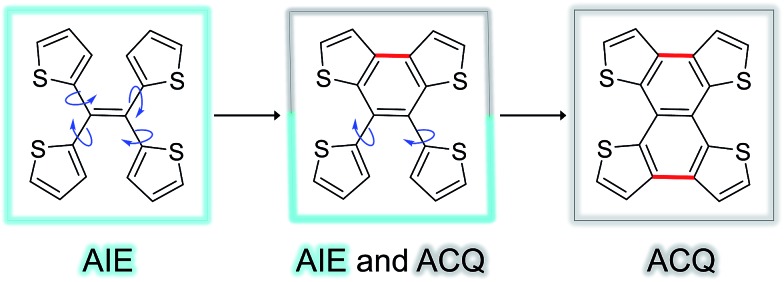
Trend of the AIE phenomenon as a function of molecular structure. The blue arrows indicate the possible rotation of the thiophene rings around the single bond.

Fig. 5 shows the complete series of TTE, sl-TTE and fl-TTE, constructed with the number of locking positions. Thanks to the rotatable parts of the four thiophenes, TTE has typical AIE characteristics. Gradually, as two of the thiophenes become locked by a covalent bond at the β positions, the molecule sl-TTE shows both AIE and ACQ properties. Eventually, when the four thiophenes are locked into a planar structure, the molecule suffers from the ACQ effect. This reinforces the theory behind the AIE mechanism where rotatable moieties non-radiatively dissipate excited-state energy. Upon aggregation, their motions become hindered and emission is enhanced. Due to the propeller-shaped molecular structure, intermolecular π–π stacking interactions are prevented, allowing for strong solid state emission. By locking the rings, the AIE property is lost as a consequence of the formation of a conventional planar luminophore that becomes afflicted with the ACQ phenomenon.

As TPE is an archetypal AIEgen that has been widely studied and due to its structural similarity to TTE, it is useful to compare the two molecules. A set of photophysical data for TPE has been included in Table 1 for comparison. The absolute quantum yield of powder TTE is 2.6%, which is much lower than the quantum yield of TPE. This is understandable as the thiophene rings in TTE are not generally considered to be excellent emitting species as the heavy sulfur atoms enhance excited state intersystem crossing. Thus, the *α*_AIE_ value of TTE is reasonably less than that of TPE.^55^ The *α*_AIE_ value is the emission enhancement defined as *α*_AIE_ = *I*_aggn_/*I*_soln_, where *I*_aggn_ is the emission intensity of the aggregated state in the THF/water fraction mixture and *I*_soln_ is the emission intensity of the solution state in the pure THF solution.

**Table 1:** Summary of the optical properties of TPE, TTE, sl-TTE and fl-TTE^*a*^

	*λ*_em_ (nm)		Stokes shift (nm)	*Φ*_AF_ ^*d*^ (%)	*τ*_F_ ^*d*^ (ns)	*α*_AIE_	Remark
	Aggregation	Crystal					
TPE	462	450	153	23	1.29	344	AIE
TTE	409	444	40	2.6	0.47	20	AIE
sl-TTE	380^*b*^, 389^*b*^, 489^*c*^		60^*e*^			1.47	AIE and ACQ
fl-TTE	381^*c*^		3			0.1	ACQ
DTE	401^*c*^	438	59	15	0.85	0.25	ACQ^*f*^

^*a*^ *λ*_em_ = first photoluminescence (PL) peak; *Φ*_AF_ = absolute fluorescence quantum yield; *τ*_F_ = fluorescence lifetime; *α*_AIE_ = PL intensity peak aggregation/PL intensity peak solution.

^*b*^Peak with AIE characteristics.

^*c*^Peak with ACQ characteristics.

^*d*^Measurement performed in powder state.

^*e*^Calculated considering the first emission peak, it is worth noting that the emission of this molecule is a dual emission, and the first and second emission peaks are ascribable to the emission of the thiophene rings, as explained in the manuscript.

^*f*^DTE is an ACQ molecule as the PL intensity decreases upon aggregation (see Fig. SI5 in ESI) but it is also emissive in powder form (see quantum yield and lifetime values in table herein) and in the crystal state (see Fig. SI6 in ESI).

The maximum absorption of TTE is more red-shifted than in TPE. This is reasonable because the thiophene rings are more electron-rich than the phenyl rings, since the same number of π-electrons are delocalized over 5 atoms instead of 6, respectively. As a consequence, TTE has an energy gap (both optical – see Table SI1 in ESI† – and electrochemical^48^) that is smaller than in TPE.^48^ However this behaviour is not mirrored in the photoluminescence, because the emission maxima of TTE in the THF/water aggregates and crystal state are consistently more blue-shifted than TPE (Table 1, see Fig. SI1† for aggregate PL comparison). Why do these molecules have such different luminescence properties while sharing structural similarities?

### Crystal analysis

**Fig. 6:**
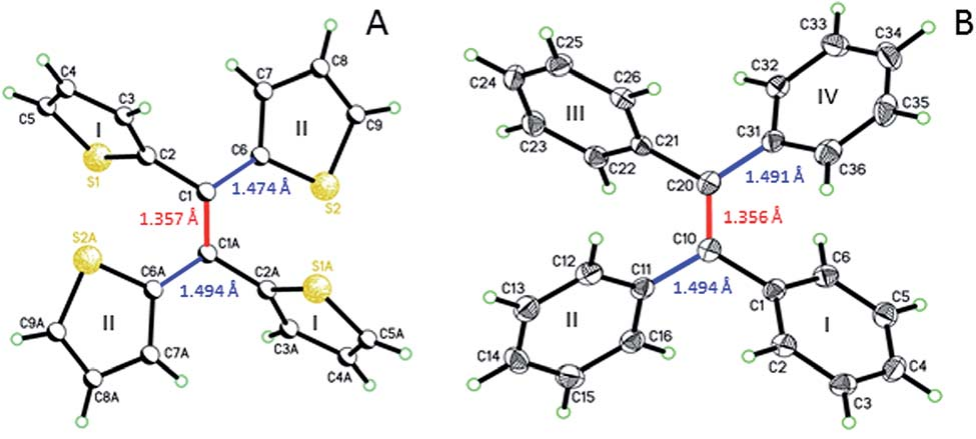
ORTEP figures of (A) TTE and (B) TPE, where the double and single bond lengths are highlighted.

Single crystal analysis of TTE (Fig. 6) allowed us to study the molecule in further detail. The comparison between the crystal structures of TTE and TPE^56^ provides a precious opportunity to better understand the emission behaviour of TTE and why the emission is regularly more blue-shifted than TPE. The measured dihedral angles between each ring and the central double bond plane for both TPE and TTE are listed in Table 2. Unlike the TPE, TTE does not adopt a propeller-shaped structure. Neither are the dihedral angles of TTE similar to those of TPE. Moreover, the highly symmetrical structure of TTE turns out to have two pairs of identical dihedral angle values of 24.61° and 84.72°, respectively. The smaller angle value of 24.61° suggests that two of the thiophenes in TTE are better conjugated with the double bond centre than the phenyl rings of TPE, which have dihedral angles between 45° to 56°. The other angle of 84.72° indicates there are two thiophenes which are nearly perpendicular to the double bond plane and thus have minimal electronic communication with the central double bond and the other thiophene rings.

**Table 2:** Dihedral angles^*a*^ of TTE and TPE

Aromatic ring^*b*^	TTE	TPE
I	84.72	45.50
II	24.61	47.33
III	—	44.59
IV	—	56.37

^*a*^Angle between the plane of each ring and the one of the double bond (see example in Fig. SI4 ESI).

^*b*^Aromatic rings for both molecules are labelled in Fig. 6.

**Fig. 7:**
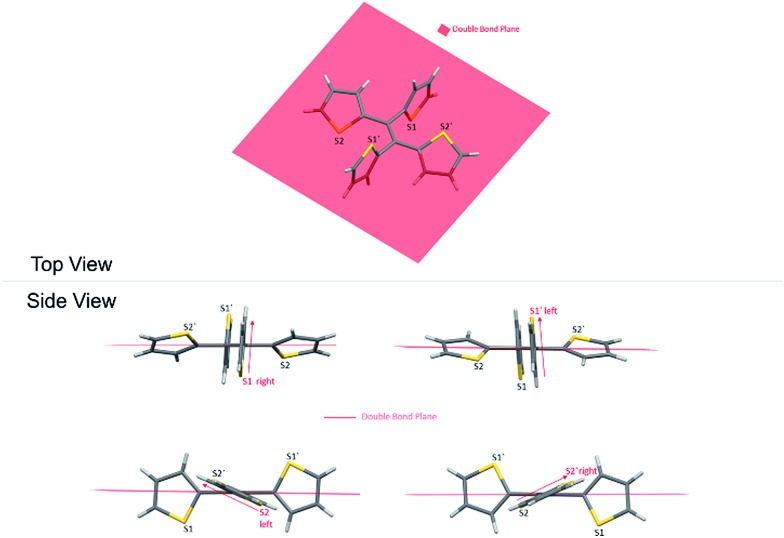
Top and side view of the orientation of the thiophene rings with respect to the double bond.

As a result, the crystal structure of TTE is quite different to the structure of TPE. The thiophene rings of TTE can be resolved into two mentioned sets: (i) the orthogonal set of thiophenes has the sulfur atom labelled S1 and S1′; and (ii) the more conjugated set of thiophene rings with S2 and S2′ (Fig. 7). When the double bond plane is placed horizontally, the S1 and S1′ thiophene rings can be seen adopting positions which are parallel to each other (Fig. 7, side view). When the thiophene with the S1 atom is closest to the viewer, the thiophene ring is tilted to the right (“right-handed”); and when the S1′ ring is closest to the readers, the ring is tilted to the left (“left-handed”). The same phenomenon is also observed for the S2/S2′ rings. This contrasts with the TPE molecule where all its aromatic rings have a left-handed orientation (Fig. SI2†). The two sets of thiophene rings play a large role in the conjugation of the entire system and consequently strongly influence its photophysical behaviour. The S1 and S1′ thiophene rings, due to their almost orthogonal positioning (Fig. 7), can be seen as almost being isolated from the conjugation system. On the other hand, S2 and S2′ rings are almost in the same plane as the double bond. This closely mirrors the structure of (*E*)-1,2-di(thiophen-2-yl)ethene (*trans*-dithienylethene, DTE), where the two thiophene rings lie in the same plane as the double bond (Fig. SI7 and SI8†). The emission of DTE has a maximum peak at 401 nm in the aggregated state (Fig. SI5†). Thus, the emission observed for TTE (Table 1) can be explained by emission from the backbone of S2 and S2′ thiophenes connected by the double bond, which is structurally analogous to DTE, and that the S1 and S1′ thiophenes barely contribute to the observed emission.

**Fig. 8:**
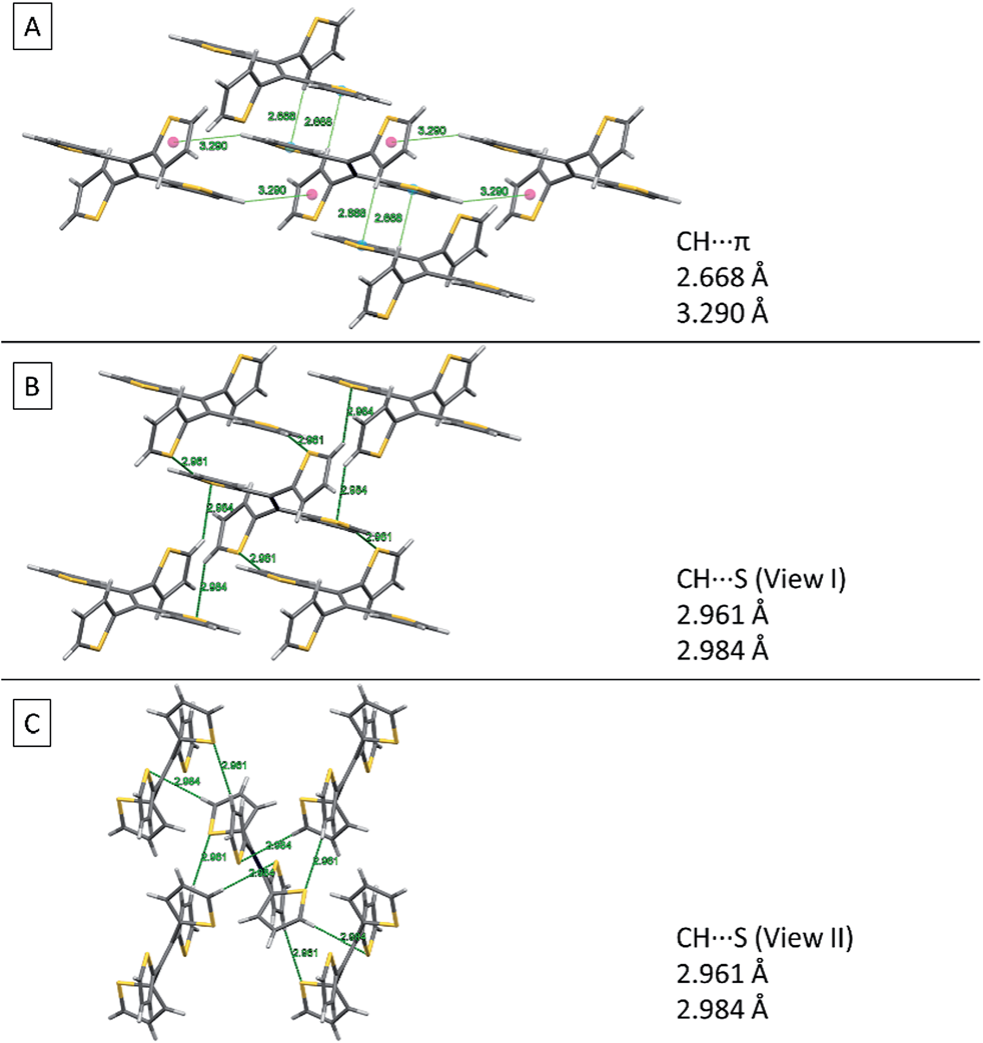
Non-covalent interactions in the TTE crystal. (A) CH···π, (B) side view and (C) top view of CH···S interactions in the TTE crystal.

Fig. 8 shows two kinds of non-covalent interaction: CH···π (Fig. 8A), and CH···S (Fig. 8B and C) in TTE crystal packing, whereas TPE only has CH···π interactions (Fig. SI3†). The CH···π interactions in TTE have lengths of 2.668 Å and 3.290 Å. The shorter distanced CH···π interaction is between one of the perpendicular thiophene hydrogens and the nearest adjacent intermolecular thiophene unit, which lies in the same plane as the double bond. Meanwhile, one of the coplanar thiophene hydrogens points to the π-system of its nearest orthogonal thiophene ring, resulting in a CH···π interaction with a distance of 3.290 Å (Fig. 8A). The CH···S interactions have measured distances of 2.961 Å and 2.984 Å. One of the CH···S interactions exists between the perpendicular thiophene sulfur atom and its nearest intermolecular aromatic hydrogen at a distance of 2.961 Å. In addition, the sulfur of the thiophene ring lying coplanar to the double bond interacts with its adjacent hydrogen at a length of 2.984 Å. Such interactions exist abundantly in the TTE crystal packing and together these interactions rigidify and stabilize the packing, preventing the free rotation of each thiophene ring. Thus, non-radiative relaxation becomes blocked and fluorescence can be observed in the crystal *via* radiative decay.

As has been reported for hexaphenylsilole (HPS), PL behaviour can show a dependency on the aggregation state of the luminogen in a phenomenon called: “morphochromism”.^55,57^ Comparing the emission spectrum of TTE for both its THF/water aggregates and crystals (Fig. 2), the peak for the crystal emission is red-shifted from its aggregation state. However, this type of morphochromism is atypical for AIEgens, where the crystal emission is usually blue-shifted from its aggregation state. In typical AIEgens, crystal structures show that the aromatic rings usually adopt a highly twisted conformation due to the packing. High dihedral angles reduce electronic communication in the π system, which likely results in the more blue-shifted emission. TPE exemplifies this perfectly, whose emission from its THF/water aggregates occurs at 462 nm while the crystal state emission is blue-shifted to 455 nm. In contrast, the THF/water aggregate sample of TTE emits at 409 nm while its crystal state emission is red-shifted to 444 nm, as shown in Fig. SI33.† As can be seen from powder X-ray diffraction (pXRD) analysis, the aggregate formed from a THF/water mixture is amorphous in nature, lacking any distinctive crystal peaks. Why does TTE have an opposite morphochromic behaviour although it is a typical AIEgen?

### S···S interactions

**Fig. 9:**
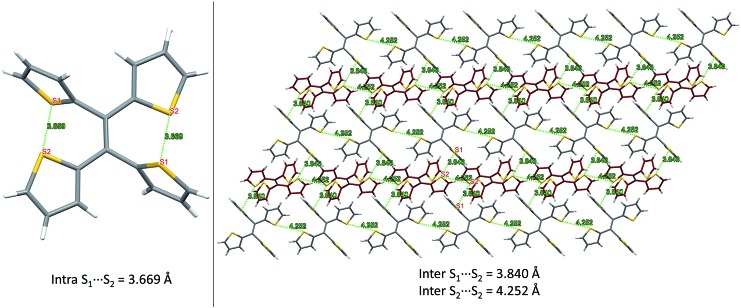
S···S interactions in the TTE crystal. Left: intramolecular interactions in the asymmetric unit of the TTE crystal; right: intermolecular interactions in the TTE crystal network.

From our previous studies of non-conventional luminescent systems, the clusteroluminogenic effect, as proposed by our group,^12^ can be one of the causes that results in the observed luminescence for systems that lack conventional π-conjugated moieties but are rich in heteroatoms. Such an effect has been primarily observed in systems with an abundance of oxygen atoms,^36^ such as carbonyl and ester functional groups. The well-aligned oxygen atoms and the intensive interactions between the oxygens that have been found in the theoretical packing of the polymer chains, are the major factors that are thought to be responsible for the clusteroluminogenic effect. Inspired by our previous clusteroluminogenic studies, we have further investigated the existence of S···S interactions (Fig. 9) in TTE. It was exciting to discover the existence of both intermolecular and intramolecular S···S interactions.

Firstly, due to the symmetry of the crystal structure there are two identical intramolecular S···S interactions between the S1 and S2 atoms distanced at 3.669 Å (Fig. 9A). Secondly, there are two different kinds of intermolecular S···S interactions: S1···S2 and S2···S2 with distance values of 3.840 Å and 4.252 Å, respectively. These interactions are abundantly interlaced throughout the TTE crystal packing. To more clearly illustrate these interactions, Fig. 9B shows a layer of the same packing consisting of rows of TTE molecules. S2 to S2 intermolecular interactions are found between the TTE molecules with the same coloured backbone; in other words, between red/red or grey/grey carbon skeletons with a distance of 4.252 Å. S1 to S2 intermolecular interactions are found between red carbon skeletons and grey carbon skeletons with distances of 3.840 Å. This crystallization-induced red-shifted emission is also observed in DTE (Fig. SI6†). The DTE crystals show a 37 nm emission red-shift with respect to the aggregate state (Table 1); its crystals have an emission maximum at 438 nm and its aggregates emit at 401 nm. It is remarkable that this red-shift is of the same magnitude as that of TTE, which has a red-shift of 35 nm. Examining the packing of the DTE crystals, similar S···S interactions are also seen (Fig. SI12†). The intermolecular S···S distances of 3.679 Å are comparable to those of TTE (3.669 Å). Shorter intramolecular and intermolecular S···S interactions have also been found in both the sl-TTE and fl-TTE crystals. In sl-TTE, the intramolecular S···S interactions have distances of 3.602 Å and 3.463 Å, whereas the intermolecular interactions have a distance of 3.513 Å (Fig. SI19†). Further examination of the published fl-TTE crystal^58^ revealed that it has intramolecular S···S distances of 3.062 Å and 3.077 Å, and intermolecular S···S distances of 3.425 Å (Fig. SI28†). It is also worth noting that although fl-TTE is a classic ACQ dye, it is emissive in the powder state (Fig. SI20 and 21†). The reason could lie in the existence of the S···S interactions, where the non-bonding through-space interaction might extend electronic communication, and create a radiative channel for dissipating energy.

**Fig. 10:**
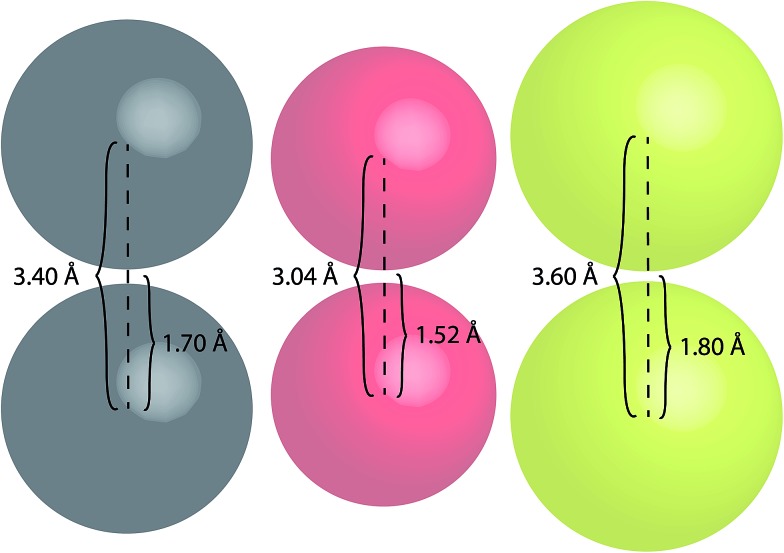
Space-filling models of carbon (grey), oxygen (red) and sulfur (yellow) atoms illustrating their van der Waals radii and centre-to-centre distances.

To better highlight the significance of these S···S distances, a comparison between the van der Waals radii of several atoms has been made (Fig. 10).

Sulfur atoms have a van der Waals radius of 1.80 Å. Based on this, the inter-atomic distances between two sulfur centres would measure 3.60 Å (Fig. 10); this highlights the proximity of the intramolecular and intermolecular distances for crystal forms of both TTE (3.669 Å) and DTE (3.679 Å). A similar consideration can be drawn for the PMAh system.^36^ The solution of the maleic anhydride monomer shows no visible emission while the solution of PMAh displays sky blue emission. The optimized structure of PMAh exhibited very close oxygen–oxygen distances of 3.1 Å and it was theorized that these interactions were highly significant for the observed emission. The van der Waals radius for oxygen is 1.52 Å (Fig. 10). As such, the inter-atomic distances between two oxygen centres would only be 3.04 Å. This is very close to the calculated value for PMAh. The fact that both of these systems have interactions close to the limit of their van der Waals radii mirrors another commonly known through-space interaction: π–π interactions between aromatic carbon systems. Strong π–π interactions between carbon atoms have measured distances in the vicinity of 3.4 Å to 3.5 Å, which is the same as the inter-atomic distances of carbon atoms based on its van der Waals radius (Fig. 10). Prof Cheng *et al.* have also found selenium–selenium interactions in the crystal state of naphthodiselenophene^59^ and with Se···Se distances nearing the value of the van der Waals radii of selenium atoms. For TTE and other clusteroluminogens, the exact nature of the heteroatom interaction still needs to be identified, *i.e.* how the interactions between the orbitals result in the observed change in photoluminescence. However, one thing seems apparent and that is the strength of the interaction and thus the observable change in the emission is highly dependent on the inter-atomic distances. Therefore, in the case of TTE and DTE, it is reasonable to believe that the S···S interactions play a crucial factor in the observed red-shift in the emission. One possibility could be that the sulfur–sulfur interactions are able to extend the electronic communication or perhaps stabilize the excited electronic state. The red-shifted emission in the crystal state of TTE and DTE, in other words its clusteroluminescence, is thus likely to be due to the emergence of the S···S interactions.

The S···S interactions exemplify how intra/intermolecular heteroatom interactions can lead to luminescence, offering a model that can be applied to other heteroatom rich systems such as PMAh,^36^ and can perhaps help to understand the luminescent behaviour of other clusteroluminescent systems, including starch.

In addition, due to the polarity of the sulfur atom, this likely facilitates the interactions between TTE and water molecules. As such, it is possible that when the molecules are in an aqueous environment, the water molecules insert themselves between sulfur atoms, splitting the intramolecular S···S interactions. As such, in the aggregation state, which is induced upon the addition of water, water molecules could be trapped impeding the formation of intramolecular S···S interactions, preventing the clusteroluminogenic effect. Thus, the aggregation emission is blue-shifted when compared to its crystal emission.

## Conclusion

In this work, tetrathienylethene (TTE) has been shown to be AIE active, non-emissive in solution but emits light in the aggregated, amorphous, and crystalline states. Thanks to the presence of the sulfur heteroatoms, non-covalent S···S interactions emerge in the crystalline state and are likely the cause of a red-shift in the emission when compared to the amorphous emission. Indeed, similar interactions in DTE also cause the emission to be red-shifted. These heteroatom interactions help explain the clusteroluminescence observed in systems that lack conventional π-conjugation but are rich in heteroatoms, such as poly(maleic anhydride). The conversion of AIE to ACQ activity in fully-locked TTE (fl-TTE) supports the idea that the luminescence in TTE is due to the restriction of the intramolecular rotation, and that through locking its thiophene rings fl-TTE becomes a classic ACQ luminogen. This is further evidenced in semi-locked TTE, which displays both ACQ and AIE activity. Both sl-TTE and fl-TTE reveal that locking the thiophene rings diminishes AIE activity. In other words, AIE activity can be tuned by selectively locking the thiophene rings. AIE activity is premised upon rotatable/vibratable moieties that first lead to non-radiative decay and, when such motions are restricted the non-radiative channels, become blocked, resulting in radiative decay. The quantum yield of TTE is not particularly high due to the presence of the sulfur atoms, but its emission in the aggregate or solid state is still fairly strong, and hence TTE could be used in biosensor applications, which is already under investigation. With these promising results and thanks to the ease of the functionalization of TTE^17^ we are currently investigating the development of TTE as a base for the construction of new TTE-based AIEgens for more specialized applications.
