# Supplementary material for: Aggregation-induced emission: mechanistic study of the clusteroluminescence of tetrathienylethene
Source: Chem Sci. 2017 Jan 11;8(4):2629–39. doi: 10.1039/c6sc05192h (PMC5431705; doi:10.1039/c6sc05192h)
Supplement: Supplementary file 1 [file SC-008-C6SC05192H-s001.pdf]

Electronic Supplementary Material (ESI) for Chemical Science.
This journal is © The Royal Society of Chemistry 2017

Aggregation-Induced Emission: Mechanistic Study of Clusteroluminescence of Tetrathienylethene

Lucia Viglianti,[abc†] Nelson L. C. Leung,[ab†] Ni Xie,[ab] Xinggui Gu,[ab] Herman H. Y. Sung,[b] Qian Miao,[d] Ian D. Williams,[b] Emanuela Licandro,[e] and Ben Zhong Tang[abc*]

[a] HKUST Shenzhen Research Institute, No. 9 Yuexing 1st Road, South Area, Hi-tech Park, Nanshan, Shenzhen 518057, China.
[b] Department of Chemistry, Hong Kong Branch of Chinese National Engineering Research Center for Tissue Restoration and Reconstruction, Institute for Advanced Study, Division of Biomedical Engineering, Division of Life Science, State Key Laboratory of Molecular Neuroscience, Institute of Molecular Functional Materials, The Hong Kong University of Science and Technology, Clear Water Bay, Kowloon, Hong Kong, China.
[c] Guangdong Innovative Research Team, SCUT-HKUST Joint Research Laboratory, State Key Laboratory of Luminescent Materials and Devices, South China University of Technology, Guangzhou 510640, China.
[d] Department of Chemistry, The Chinese University of Hong Kong, Shatin, New Territories, Hong Kong, China.
[e] Dipartimento di Chimica, Università degli Studi di Milano, Via Golgi 19, 20133, Milano, Italy.
*Correspondence: tangbenz@ust.hk

Supporting Information

Index

Figure SI1: Comparison between the photoluminescence spectra of TTE and TPE in THF/water f_w 99%; 3
Figure SI2: Single-crystal of TPE
Figure SI3: CH···π interaction in TPE crystal structure 4
Figure SI4: Dihedral angle between S1 ring and the double bond plane in TTE single-crystal
Figure SI5: The photoluminescence (PL) spectra of DTE in THF and THF/water mixtures with increasing water fractions (f_w) to 90%. 5
Figure SI6: The photoluminescence (PL) spectrum of DTE in aggregate, and crystal state. Excitation at 322 nm.

Figure SI7: ORTEP picture of DTE crystal. 6
Figure SI8: Molecular planes of the double bond and thiophene rings, and the related dihedral angle.
Figure SI9: Packing in DTE crystal. 7
Figure SI10: CH···π interactions in DTE crystal.
Figure SI11: CH···S interactions in DTE crystal.
Figure SI12: S···S interactions in DTE crystal. 8

Figure SI13: ORTEP picture of sl-TTE crystal. 9
Figure SI14: Angle between the molecular planes in sl-TTE crystal.
Figure SI15: Packing in sl-TTE crystal. 10
Figure SI16: intra and inter-molecular π···π interactions in sl-TTE crystal.
Figure SI17 intermolecular CH···π interactions in sl-TTE crystal. 11
Figure SI18 intermolecular CH···S interactions in sl-TTE crystal.
Figure SI19 intra and inter-molecular S···S interactions in sl-TTE crystal. 12

Figure SI20 Photograph of fl-TTE (left) and TTE (right) in powder state. 13
Figure SI21 Photoluminescence spectrum of fl-TTE in powder state.
Figure SI22 Crystal structure of fl-TTE. 14
Figure SI23 Molecular plane in the asymmetric unit of fl-TTE crystal structure
Figure SI24 Molecular plane in the unit cell of fl-TTE crystal structure. 15
Figure SI25 Packing of fl-TTE in the crystal.
Figure SI26 π···π interactions in fl-TTE crystal. 16
Figure SI27 CH···π interactions in fl-TTE crystal.
Figure SI28 S···S interactions in fl-TTE crystal. 17
Figure SI29 Molar Absorptivity of TTE in THF.
Figure SI30 Molar Absorptivity of sl-TTE in THF. 18
Figure SI31 Molar Absorptivity of fl-TTE in THF.
Figure SI32 Molar absorptivity comparison of fl-TTE, sl-TTE and TTE in THF. 19
Table S1 Absorption values and energy gap of the investigated molecules
Figure SI33 X-ray diffraction spectra of the THF/water (f_w = 90%) aggregates and crystals of TTE. 20
TTE crystal data 21
DTE crystal data 27
sl-TTE crystal Data 30
Materials and general information 36
Instruments
Experimental Procedures

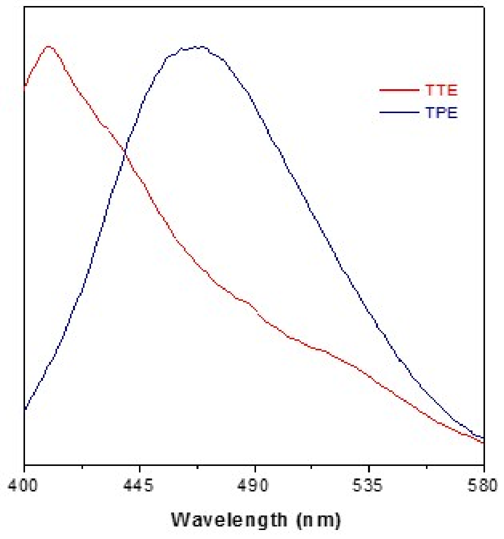

Figure SI1. The photoluminescence (PL) spectra of TTE and TPE in THF/water with 99 % of water fractions.

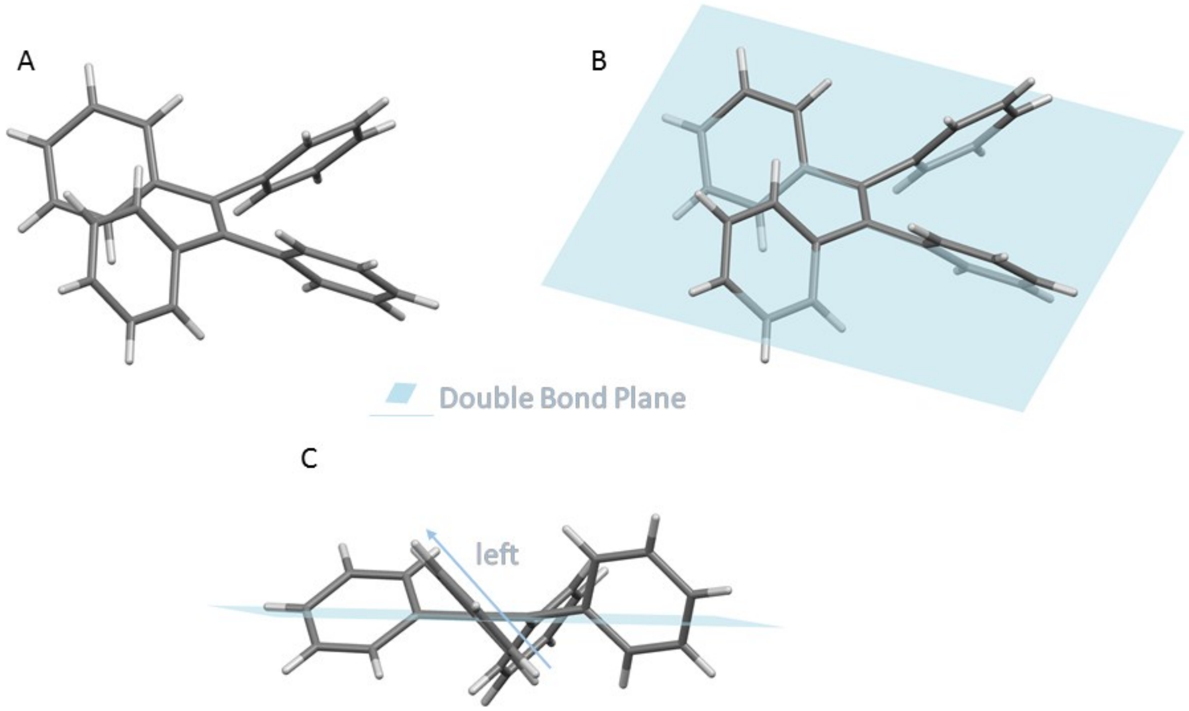

Figure SI2. Single-crystal of TPE: (A) general view, (B) top view, and (C) side view of ring direction with respect to the double bond plane.

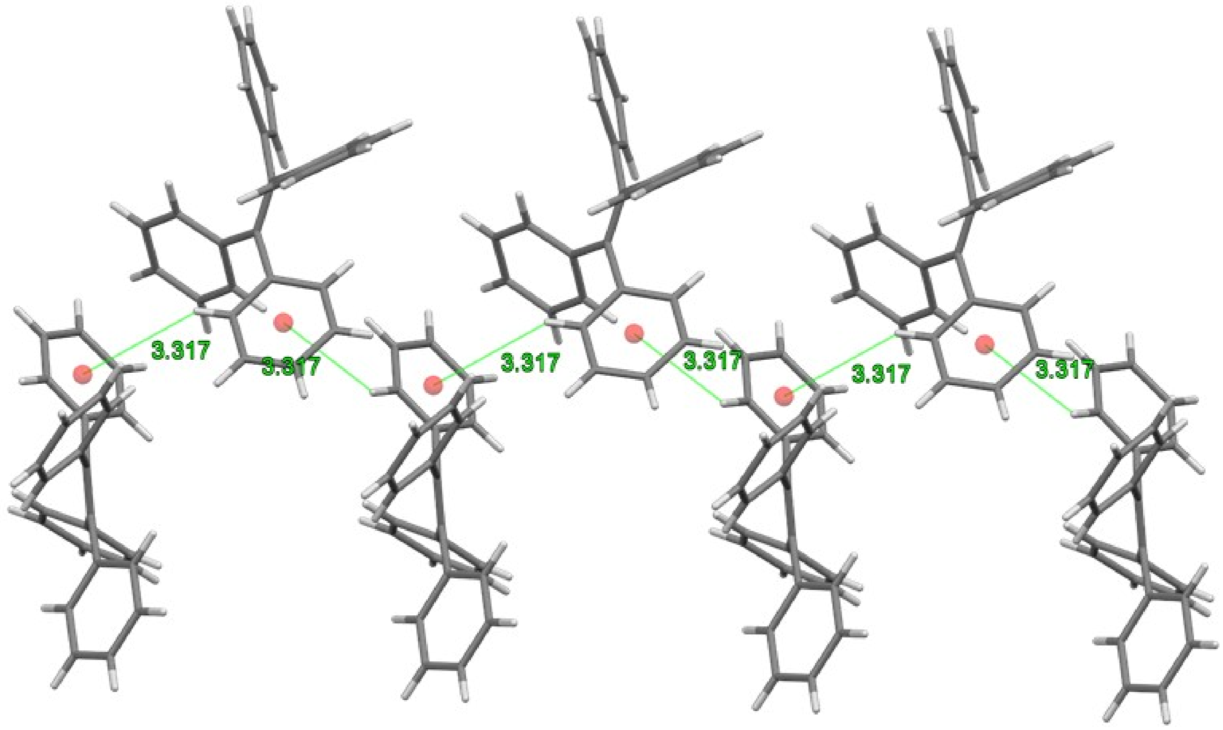

Figure SI3. CH···π interactions in TPE crystal for one of the four phenyl rings.

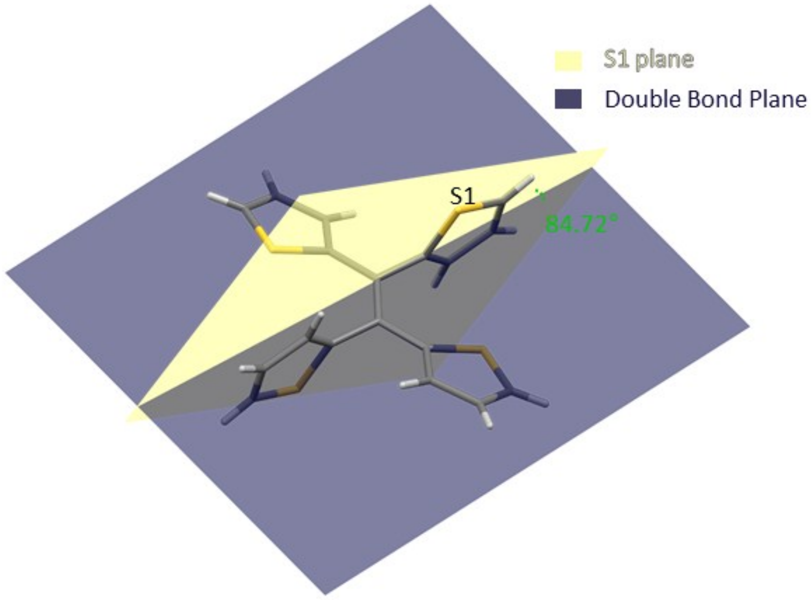

Figure SI4. Dihedral angle between the S1 ring and the double bond plane in TTE single-crystal.

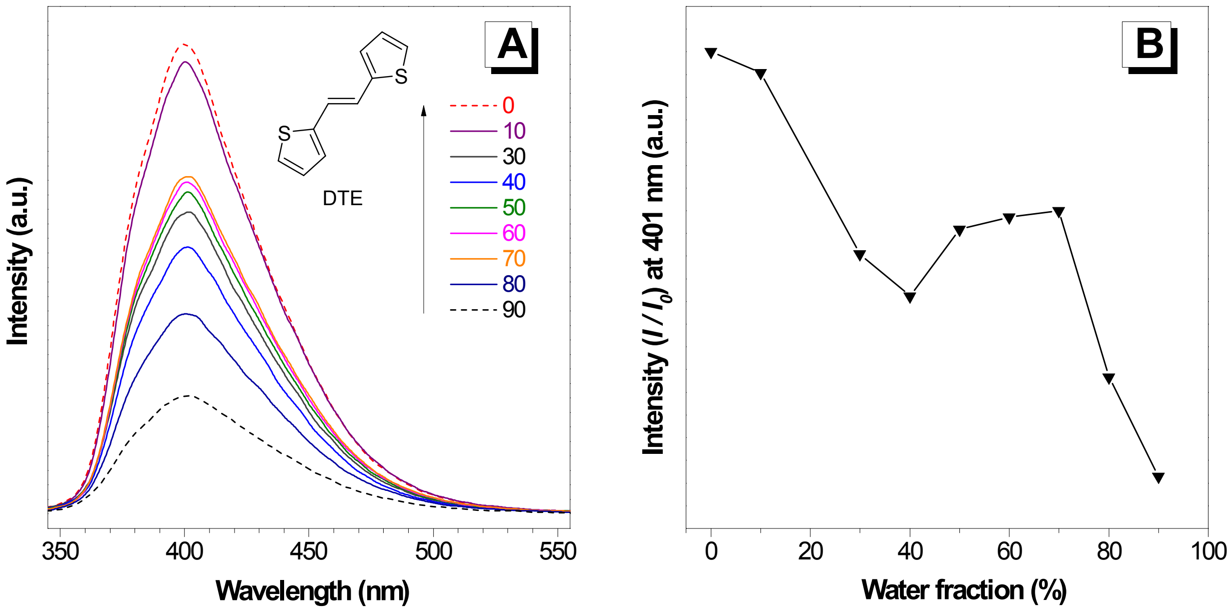

Figure SI5. (A) The photoluminescence (PL) spectra of DTE in THF and THF/water mixtures with increasing water fractions (f_w) to 90%. (B) Change in PL intensity of DTE at 401 nm versus water fraction in THF/water mixtures. Excitation at 322 nm.

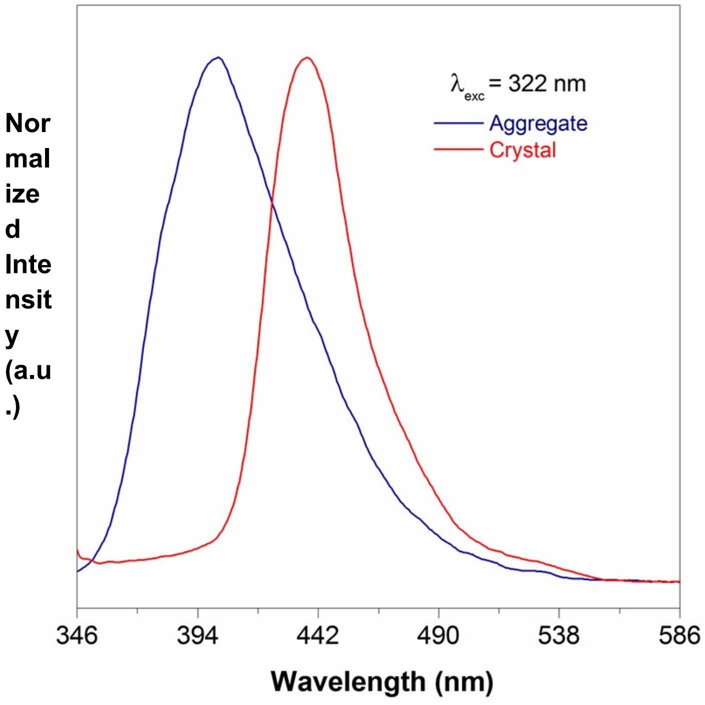

Figure SI6. The photoluminescence (PL) spectrum of DTE in aggregate, and crystal state. Excitation at 322 nm.

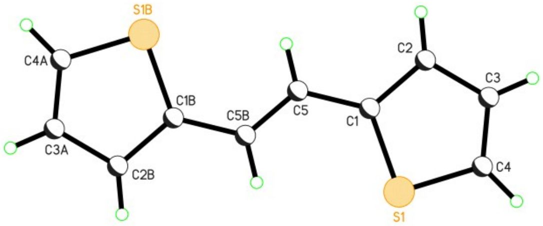

Figure SI7. ORTEP picture of DTE crystal.

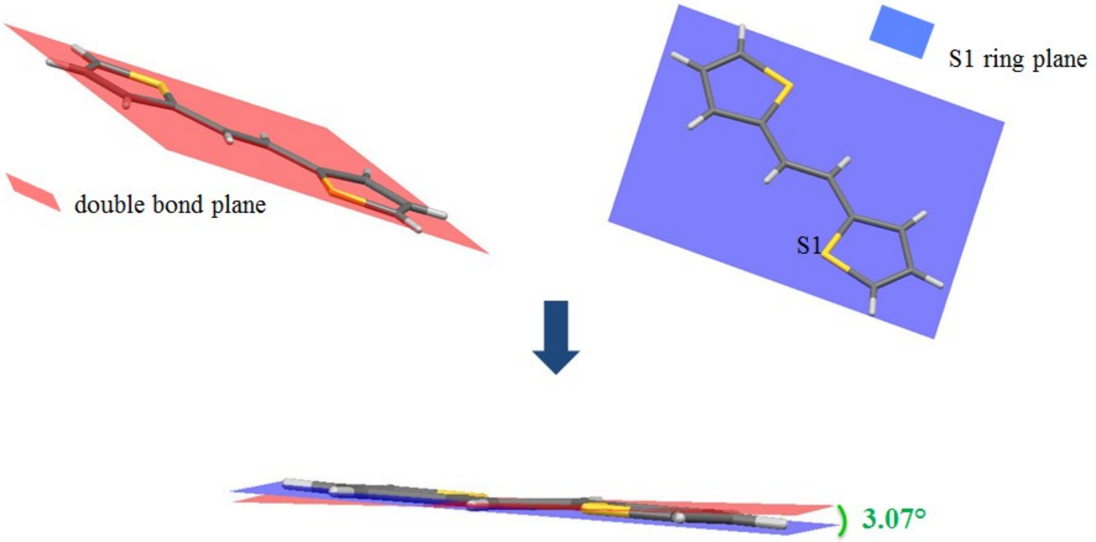

Figure SI8. Molecular planes of the double bond and thiophene rings, and the related dihedral angle.

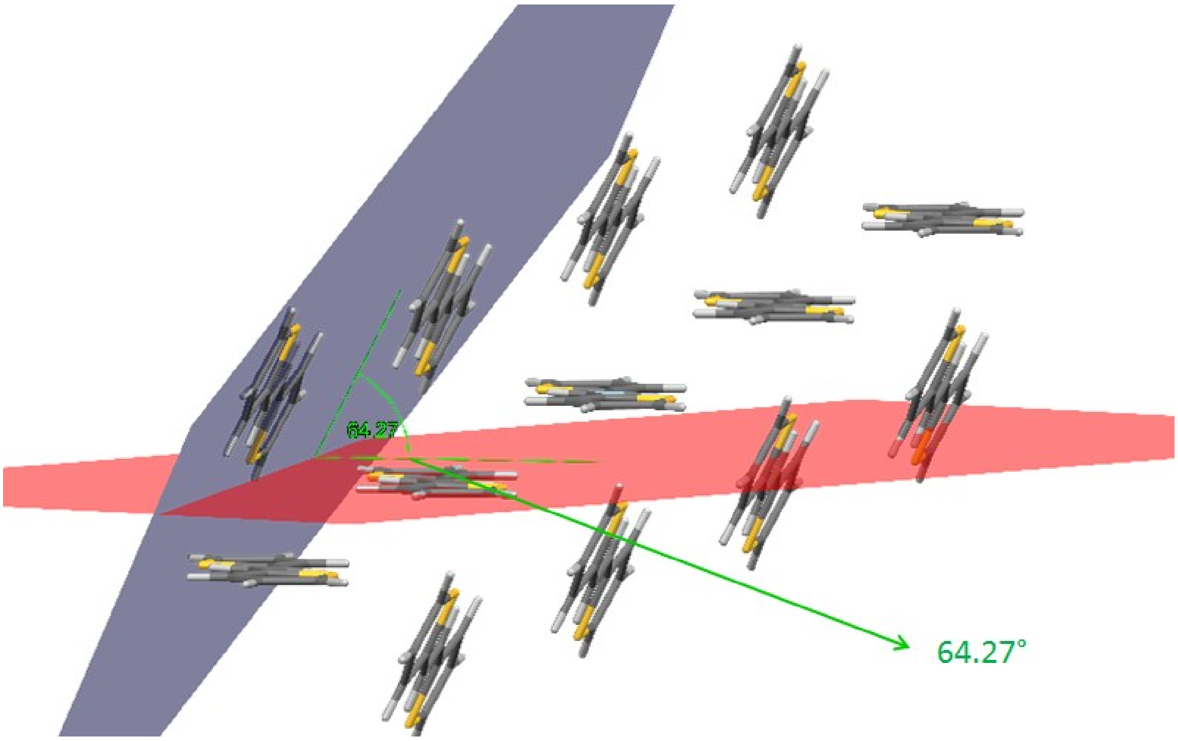

Figure SI9. Packing in DTE crystal.

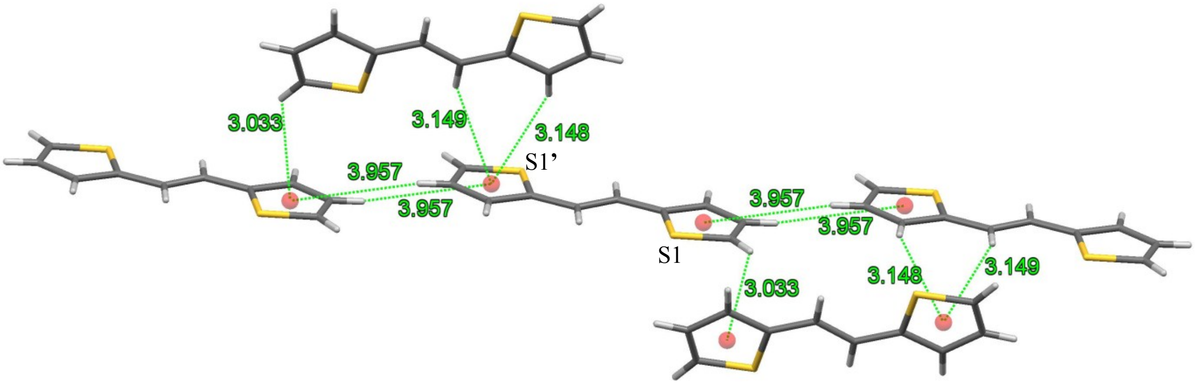

Figure SI10. CH···π interactions in DTE crystal.

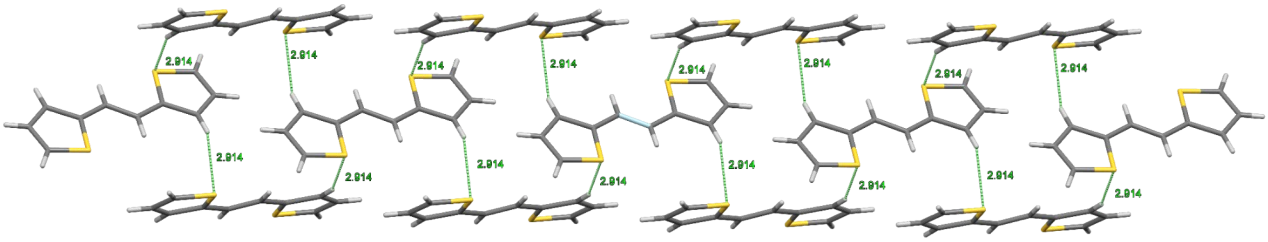

Figure SI11. CH···S interactions in DTE crystal. The measured distance is 2.914 Ấ.

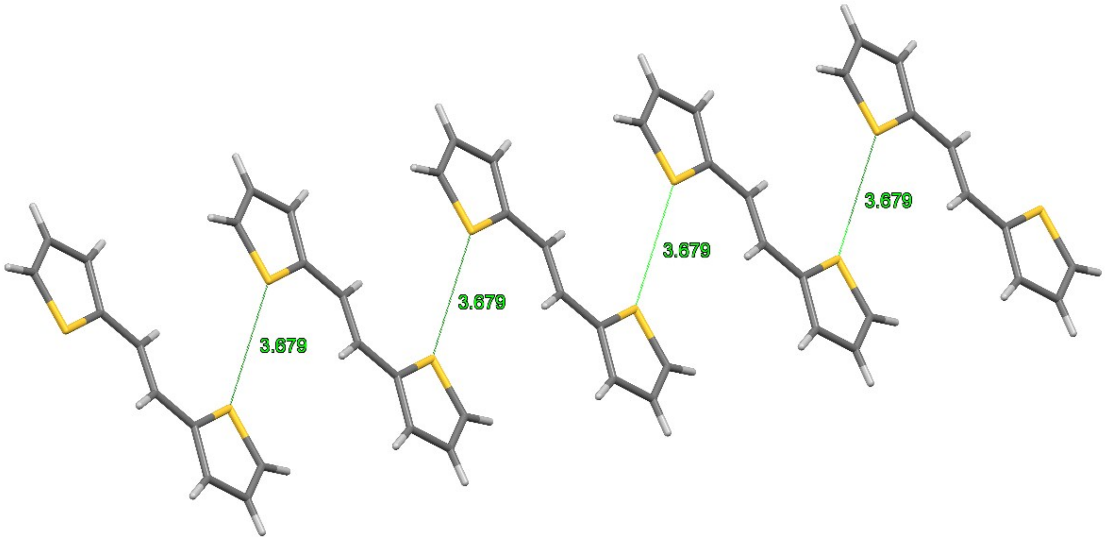

Figure SI12. S···S interactions in DTE crystal. The measured distance is 3.679 Ǻ.

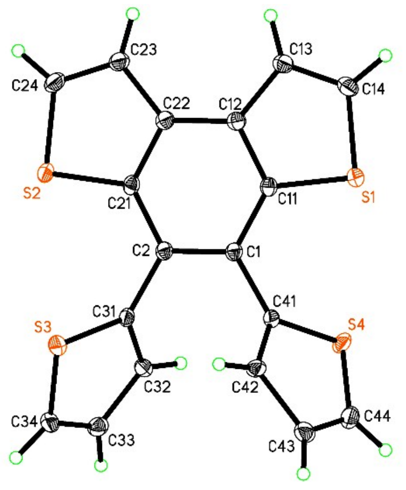

Figure SI13. ORTEP picture of sl-TTE crystal.

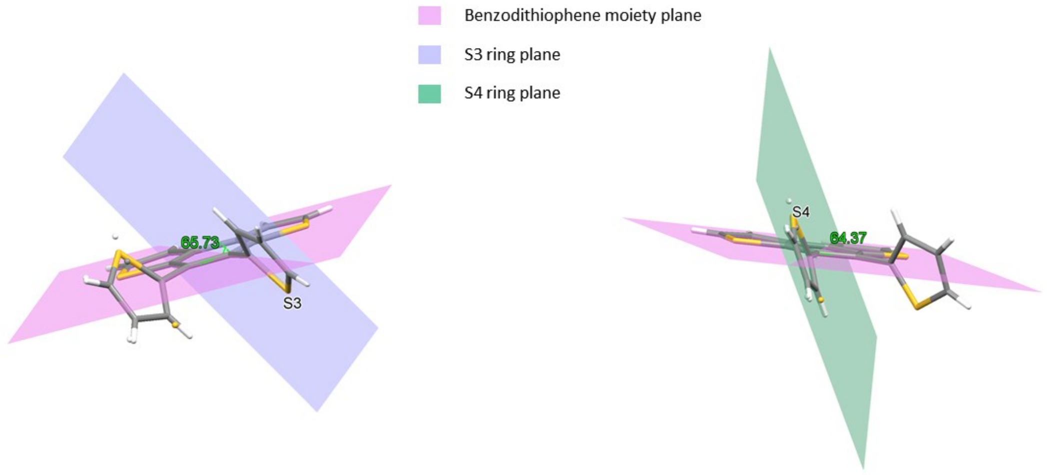

Figure SI14. Angle between S3 (left) and S4 (right) ring planes and benzodithiophene moiety plane (66˚and 64˚ respectively) in sl-TTE crystal.

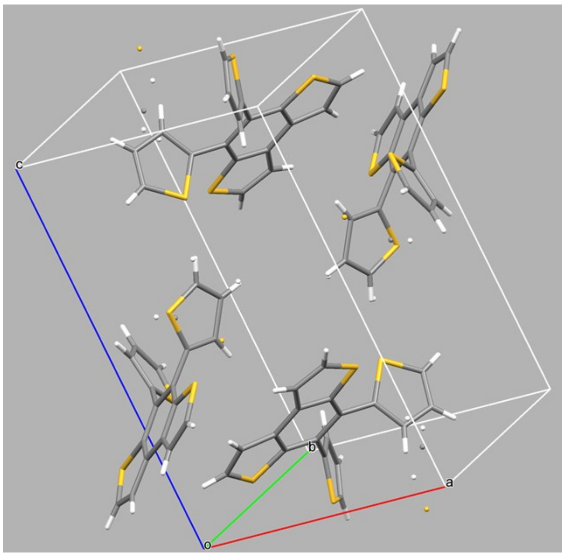

Figure SI15. Packing in sl-TTE crystal.

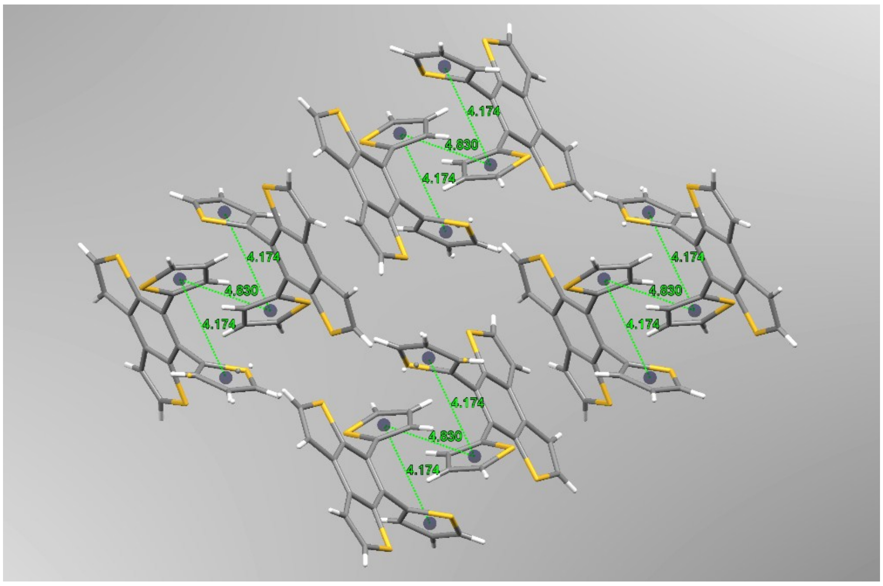

Figure SI16. *intra* and *inter*-molecular π···π interactions in sl-TTE crystal.

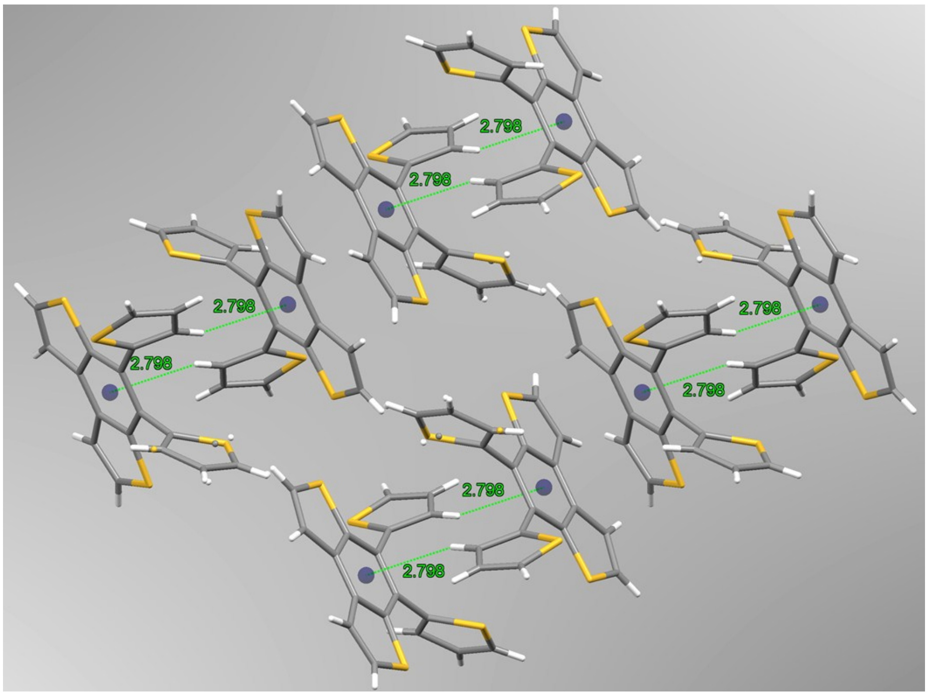

Figure SI17. *inter*molecular CH···π interactions in sl-TTE crystal.

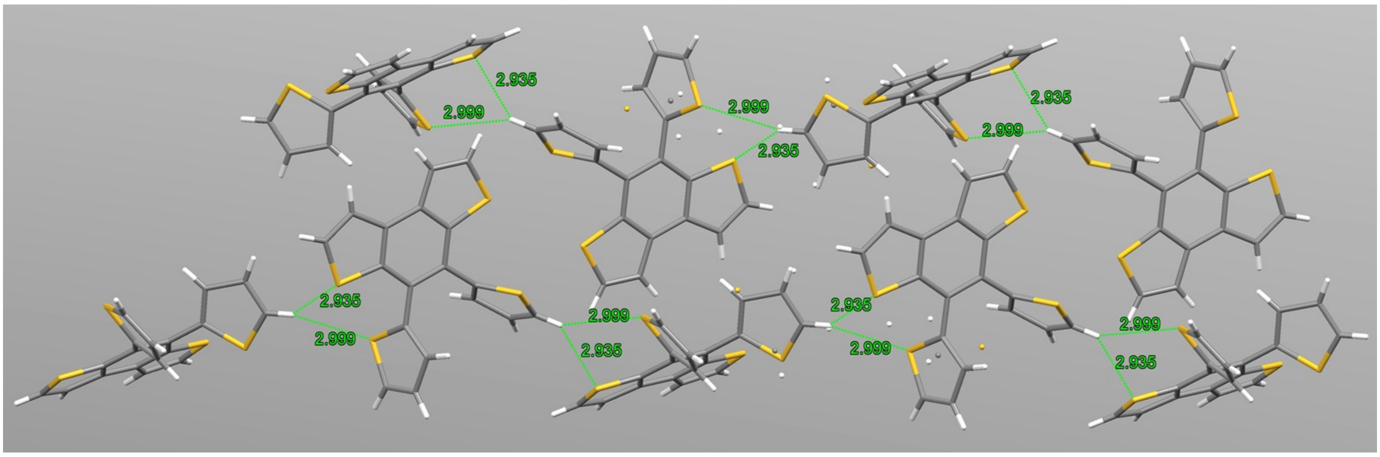

Figure SI18. *inter*molecular CH···S interactions in sl-TTE crystal.

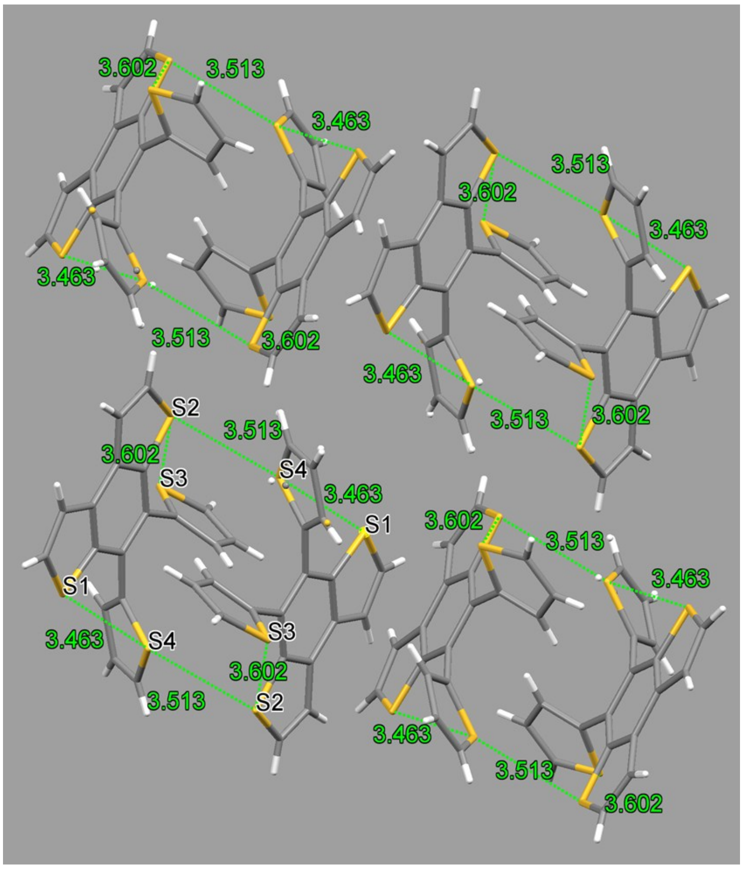

Figure SI19. *intra* and *inter*-molecular S···S interactions in sl-TTE crystal.

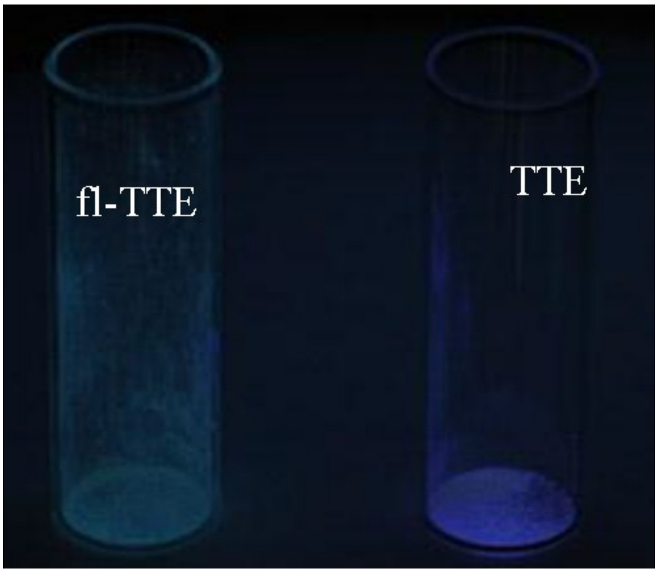

Figure SI20. Photograph of fl-TTE (left) and TTE (right) in powder state, taken under 365 nm UV-light illumination.

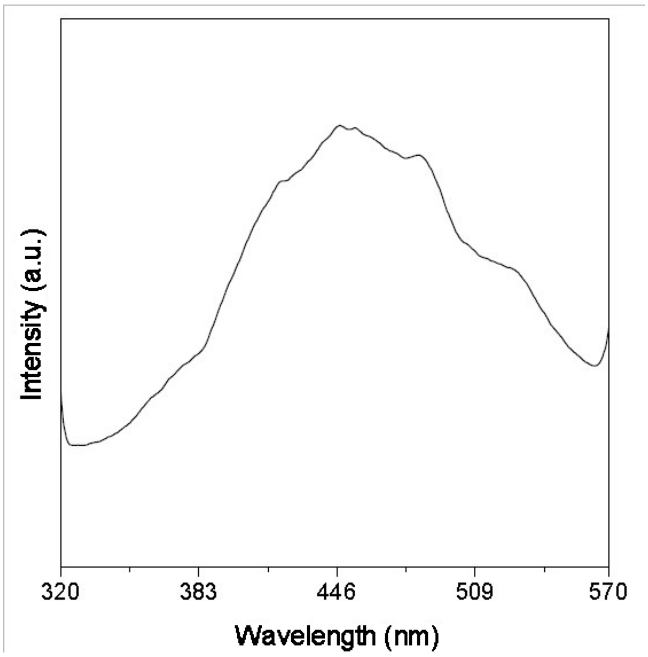

Figure SI21. Photoluminescence spectrum of fl-TTE in powder state.

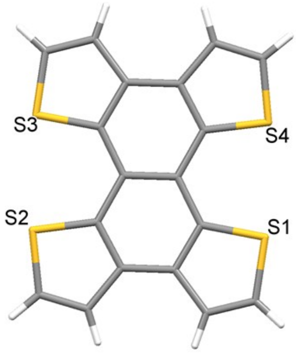

Figure SI22. Crystal structure of fl-TTE.

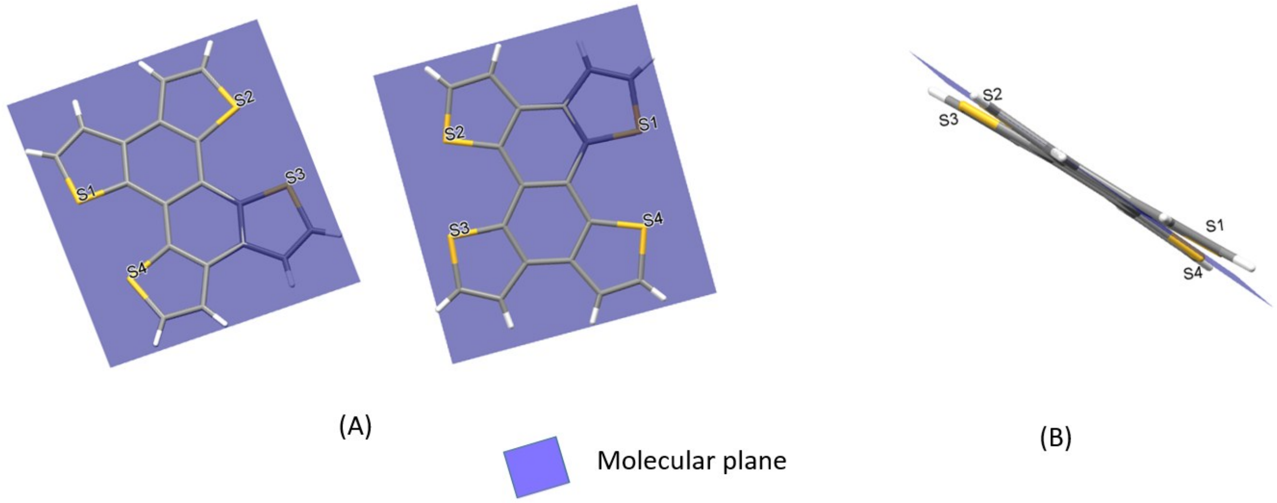

Figure SI23. Molecular plane in the asymmetric unit of fl-TTE crystal structure. S1 and S3 are out of the plane. (A) top views, (B) side view.

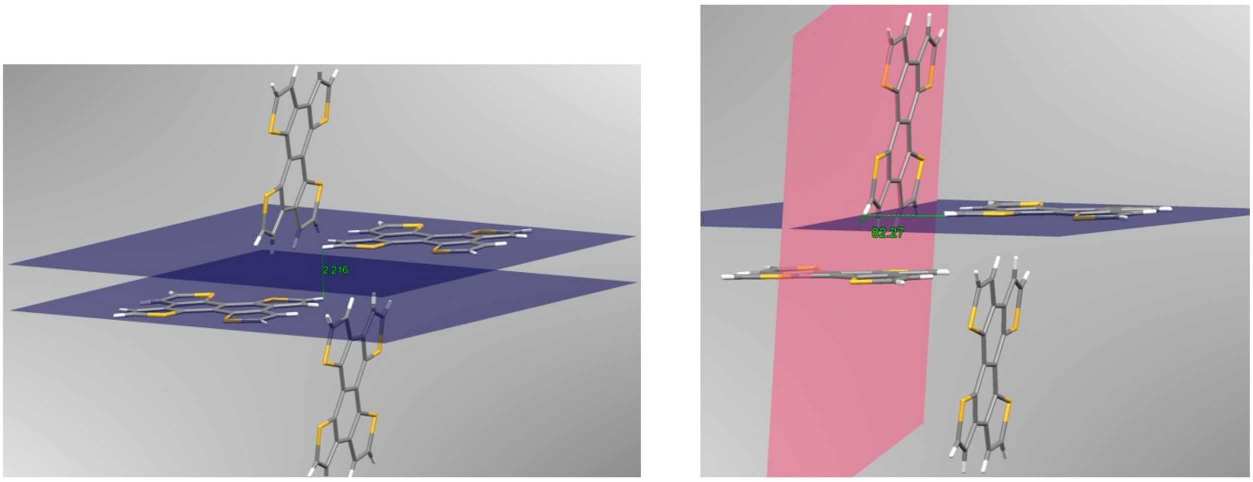

(A) (B)

Figure SI24. Molecular plane in the unit cell of fl-TTE in the crystal structure. (A) Horizontal planes (distance 2.216 Å), (B) intersection between horizontal and vertical plane (angle 83°).

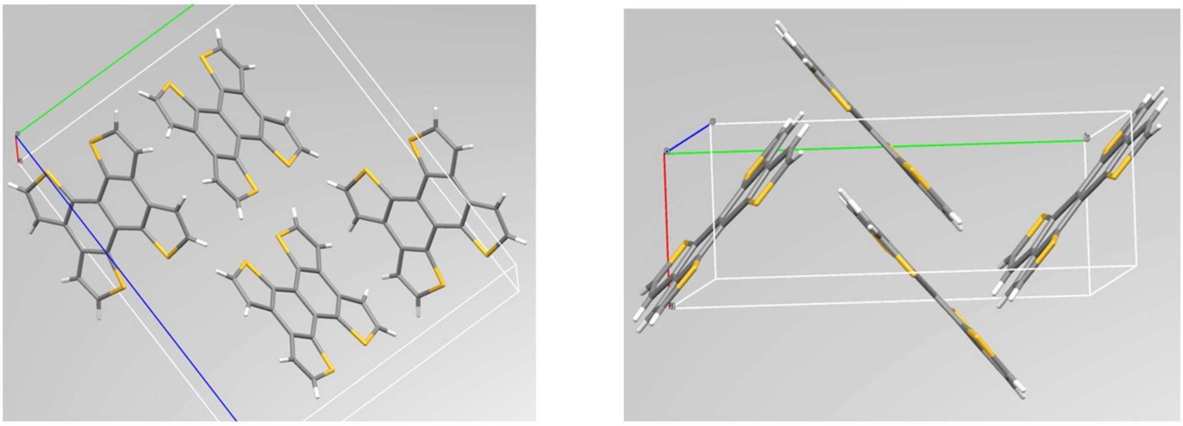

(A) (B)

Figure SI25. Packing of fl-TTE crystal. (A) top view, (B) side view.

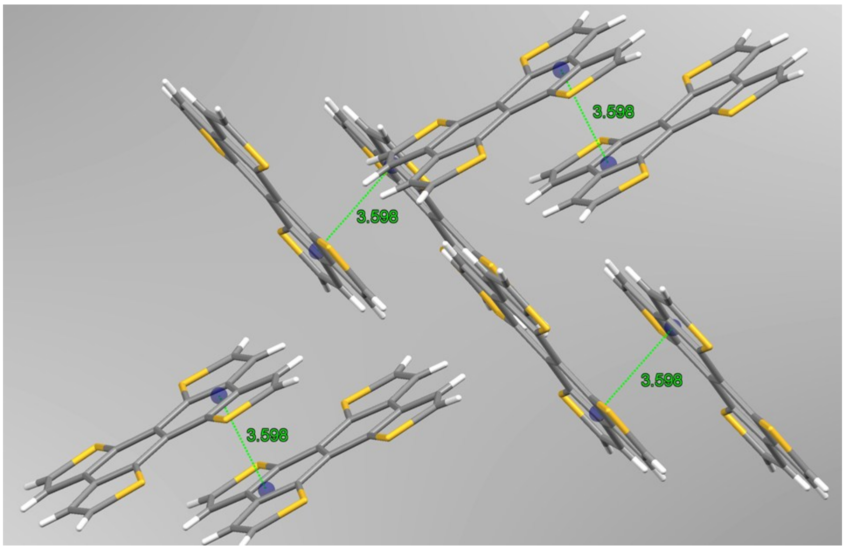

Figure SI26. π···π interactions in fl-TTE crystal.

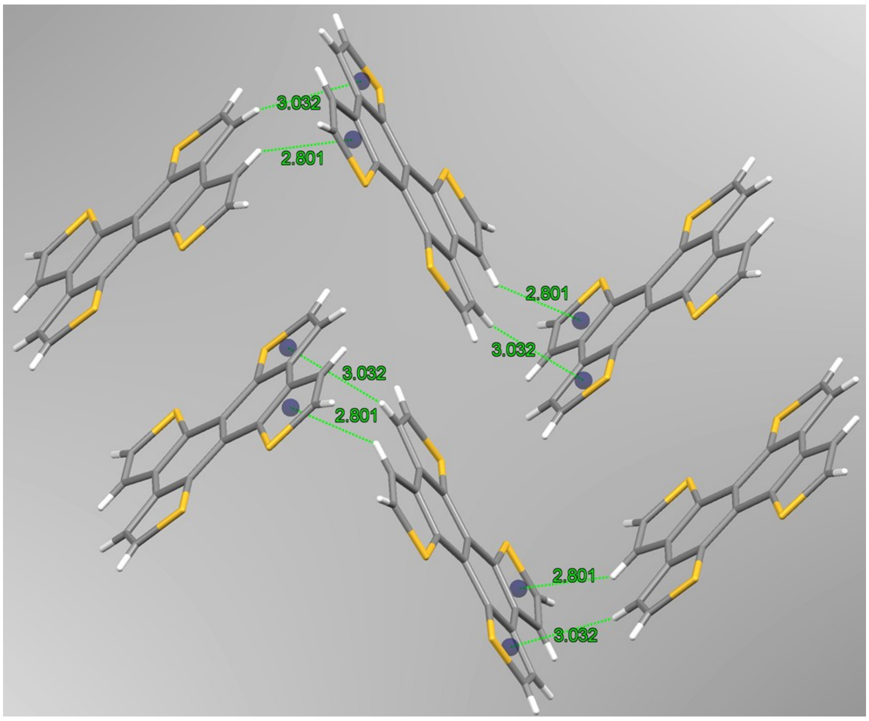

Figure SI27. CH···π interactions in fl-TTE crystal.

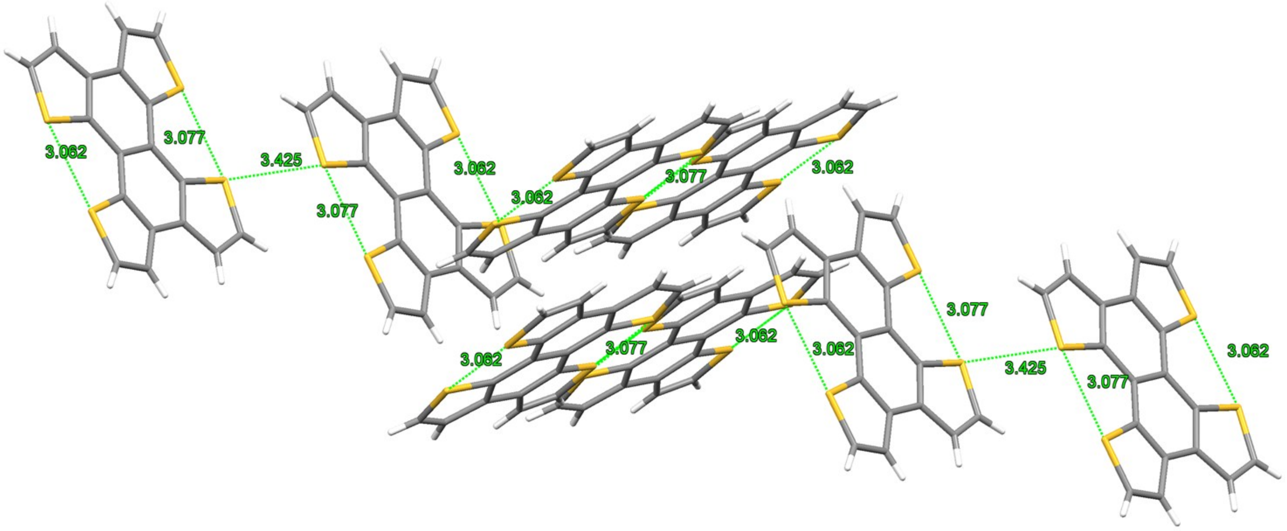

Figure SI28. S···S interactions in fl-TTE crystal.

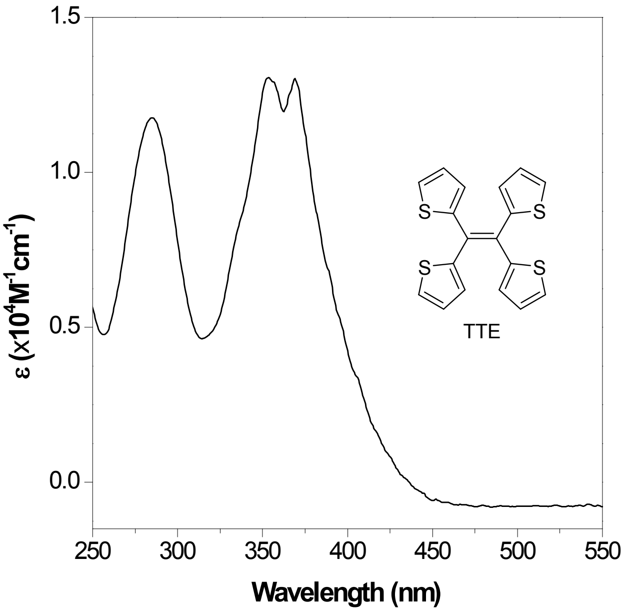

Figure SI29. Molar Absorptivity of TTE in THF.

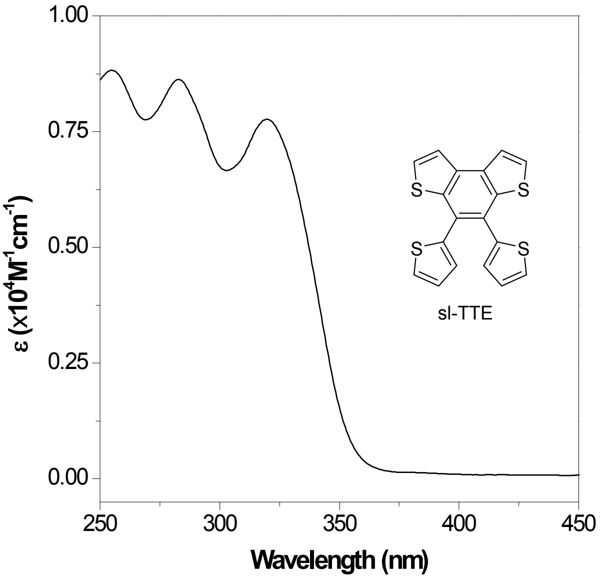

Figure SI30. Molar Absorptivity of sl-TTE in THF.

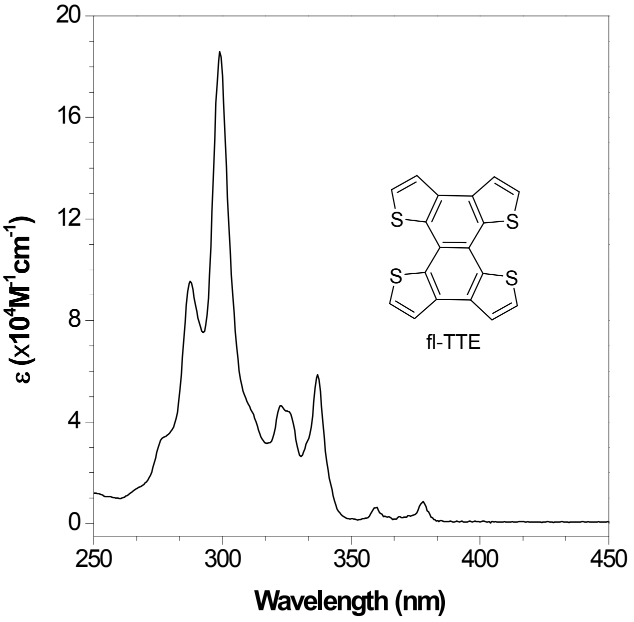

Figure SI31. Molar Absorptivity of fl-TTE in THF.

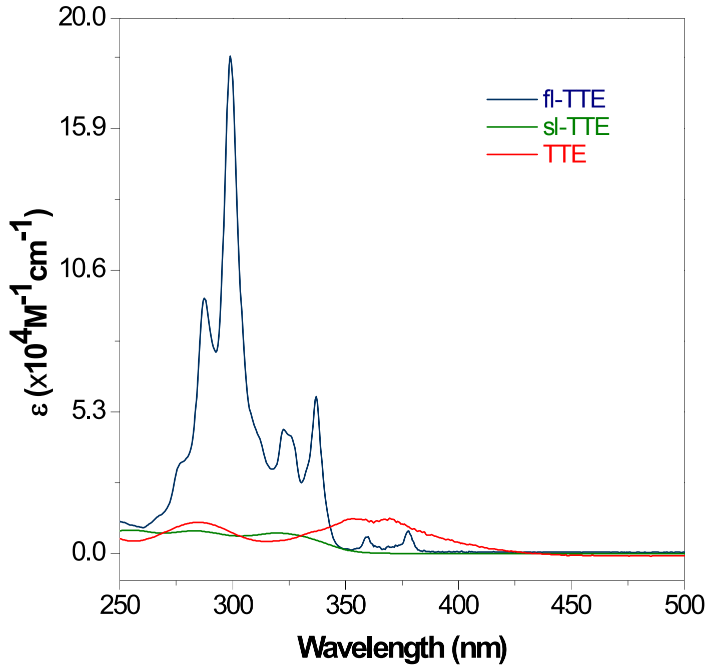

Figure SI32. Molar absorptivity comparison of fl-TTE, sl-TTE and TTE in THF.

Table SI1. Absorption values and energy gap of the investigated molecules: TPE, TTE, sl-TTE, fl-TTE and DTE.

	λ_{abs} (nm) onset values	λ_{abs} (nm) peak values	Optical Energy Gap (eV) from onset values	Optical Energy Gap (eV) from peak values
TPE	360	308	3.45	4.03
TTE	413	364	3.01	3.41
sl-TTE	359	320	3.46	3.88
fl-TTE	382	378	3.25	3.28
DTE	377	344	3.29	3.61

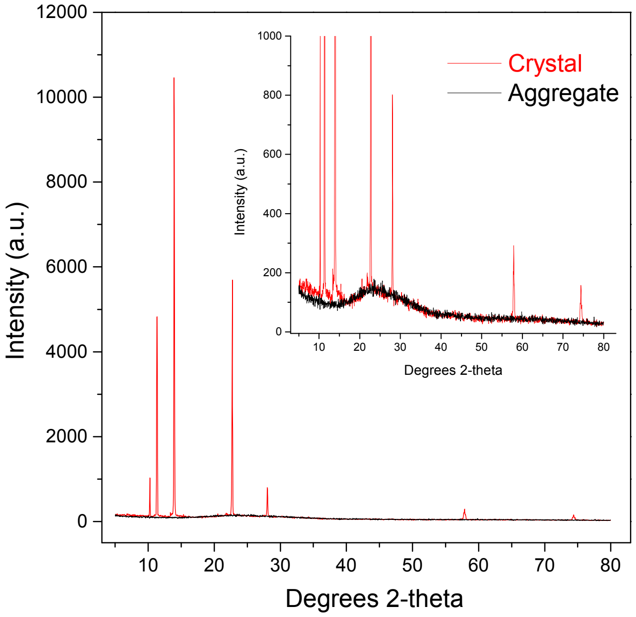

Figure SI33. X-ray diffraction spectra of the THF/water (f_w = 90%) aggregates and crystals of TTE. Inset: enlarged spectra for clarity. The THF/water aggregates are amorphous in nature.

TTE Crystal Data

**Table SI2 Crystal data and structure refinement for TTE.**	
Identification code	TTE
Empirical formula	$C_{18}H_{12}S_4$
Formula weight	356.52
Temperature/K	100.0(4)
Crystal system	monoclinic
Space group	$P2_1/n$
a/Å	9.3160(2)
b/Å	9.10329(18)
c/Å	9.6346(2)
α/°	90.00
β/°	110.853(3)
γ/°	90.00
Volume/Å^3	763.55(3)
Z	2
ρ_{calc}g/cm^3	1.551
μ/mm^{-1}	5.633
F(000)	368.0
Crystal size/mm^3	0.25 × 0.22 × 0.17
Radiation	CuKα (λ = 1.54184)
2Θ range for data collection/°	11.34 to 133.94
Index ranges	$-10 \leq h \leq 11$, $-10 \leq k \leq 7$, $-11 \leq l \leq 11$
Reflections collected	3875
Independent reflections	1353 [R_{int} = 0.0145, R_{sigma} = 0.0151]
Data/restraints/parameters	1353/2/108
Goodness-of-fit on F^2	1.000
Final R indexes [I>=2σ (I)]	R_1 = 0.0250, wR_2 = 0.0641
Final R indexes [all data]	R_1 = 0.0261, wR_2 = 0.0648
Largest diff. peak/hole / e Å^{-3}	0.38/-0.22

Table SI3 Fractional Atomic Coordinates (×10⁴) and Equivalent Isotropic Displacement Parameters (Å²×10³) for TTE. U_{eq} is defined as 1/3 of the trace of the orthogonalized U_{IJ} tensor.				
**Atom**	***x***	***y***	***z***	**U(eq)**
S1	2587.0(4)	1554.3(4)	4207.7(4)	14.32(13)
S2	1028.6(5)	-22.1(5)	8353.0(5)	16.62(14)
S2A	814(18)	2853(14)	7092(15)	9(3)
C1	332.7(18)	648.3(19)	5289.3(18)	11.7(3)
C1A	10(30)	-160(30)	5740(30)	9(5)
C2	797.9(18)	1722.3(17)	4355.5(18)	14.0(3)
C3	42.1(19)	2949.1(18)	3614.2(18)	15.4(3)
C4	929.8(19)	3721.6(18)	2902.8(18)	14.7(3)
C5	2313.3(19)	3097.3(18)	3131.7(18)	15.2(3)
C6	693.9(17)	1120.4(18)	6840.4(18)	13.3(3)
C7	910(5)	2589(5)	7298(5)	15.9(8)
C8	1309.4(19)	2732.2(19)	8874.5(19)	17.1(3)

C9	1428.8(19)	1413.3(19)	9560.8(19)	17.8(4)

Table SI4 Anisotropic Displacement Parameters ($Å^2×10^3$) for TTE. The Anisotropic Displacement Factor Exponent Takes the Form: $-2\pi^2 [h^2a^{*2}U_{11}+2hka^*b^*U_{12}+…]$.						
**Atom**	**U_{11}**	**U_{22}**	**U_{33}**	**U_{23}**	**U_{13}**	**U_{12}**
S1	13.3(2)	14.8(2)	17.2(2)	2.53(14)	8.29(16)	0.98(14)
S2	21.1(2)	15.9(2)	13.9(2)	1.03(16)	7.57(18)	0.87(16)
C1	10.3(8)	12.5(8)	13.8(8)	2.2(7)	6.2(6)	2.8(6)
C2	12.3(8)	15.1(8)	16.8(8)	0.1(6)	7.8(6)	-1.6(6)
C3	14.6(8)	15.4(8)	17.2(8)	1.9(6)	6.9(6)	-3.5(6)
C4	16.8(8)	13.4(8)	14.1(8)	1.5(6)	5.6(6)	-1.5(6)
C5	16.9(8)	15.3(8)	15.8(8)	1.1(6)	8.7(6)	-3.2(6)
C6	10.3(7)	16.2(8)	14.2(8)	-0.9(6)	5.3(6)	0.2(6)
C7	10.5(13)	16.5(18)	21.9(18)	1.8(13)	7.1(12)	0.1(12)
C8	14.0(8)	18.1(8)	18.7(8)	-6.4(7)	5.2(6)	-0.5(6)
C9	15.9(8)	24.0(9)	13.6(8)	-2.7(7)	5.5(7)	1.1(7)

Table SI5 Bond Lengths for TTE.

Atom	Atom	Length/Å		Atom	Atom	Length/Å
S1	C2	1.7292(16)		C1A	C2[1]	1.59(3)
S1	C5	1.7101(16)		C1A	C6	1.55(3)
S2	C6	1.7260(16)		C2	C1A[1]	1.59(3)
S2	C9	1.7005(17)		C2	C3	1.377(2)
S2A	C6	1.593(14)		C3	C4	1.432(2)
S2A	C8	1.615(14)		C4	C5	1.353(2)
C1	C1[1]	1.357(4)		C6	C7	1.400(4)
C1	C2	1.494(2)		C7	C8	1.436(5)
C1	C6	1.474(2)		C8	C9	1.356(2)
C1A	C1A[1]	1.45(5)				

Table SI6 Bond Angles for TTE.

Atom	Atom	Atom	Angle/°		Atom	Atom	Atom	Angle/°
C5	S1	C2	92.13(8)		C4	C5	S1	111.82(12)
C9	S2	C6	92.55(8)		S2A	C6	S2	119.1(5)
C6	S2A	C8	94.2(6)		C1	C6	S2	126.00(13)
C1[1]	C1	C2	120.65(19)		C1	C6	S2A	114.9(5)
C1[1]	C1	C6	125.17(19)		C1	C6	C1A	36.8(9)
C6	C1	C2	114.17(15)		C1A	C6	S2	91.7(9)
C1A[1]	C1A	C2[1]	107(2)		C1A	C6	S2A	146.9(11)
C1A[1]	C1A	C6	112(3)		C7	C6	S2	110.3(2)
C6	C1A	C2[1]	141.2(17)		C7	C6	S2A	9.0(6)
C1	C2	S1	119.12(12)		C7	C6	C1	123.6(2)
C1	C2	C1A[1]	38.7(9)		C7	C6	C1A	155.7(10)
C1A[1]	C2	S1	111.5(9)		C6	C7	C8	112.0(3)
C3	C2	S1	111.04(12)		C7	C8	S2A	9.2(6)
C3	C2	C1	129.72(15)		C9	C8	S2A	121.4(5)

C3	C2	C1A[1]	123.2(9)		C9	C8	C7	112.4(2)
C2	C3	C4	111.76(15)		C8	C9	S2	112.75(13)
C5	C4	C3	113.24(15)					

[1]-x,-y,1-z

Table SI7 Torsion Angles for TTE.									
**A**	**B**	**C**	**D**	**Angle/°**	**A**	**B**	**C**	**D**	**Angle/°**
S1	C2	C3	C4	1.32(18)	C2[1]	C1A	C6	S2A	-135(2)
S2	C6	C7	C8	-1.4(3)	C2[1]	C1A	C6	C1	-175(4)
S2A	C6	C7	C8	166(5)	C2[1]	C1A	C6	C7	-131(2)
S2A	C8	C9	S2	0.7(7)	C2	C3	C4	C5	-1.0(2)
C1[1]	C1	C2	S1	-86.5(2)	C3	C4	C5	S1	0.20(19)
C1[1]	C1	C2	C1A[1]	2.1(14)	C5	S1	C2	C1	-177.34(14)
C1[1]	C1	C2	C3	98.0(3)	C5	S1	C2	C1A[1]	140.5(10)
C1[1]	C1	C6	S2	25.5(3)	C5	S1	C2	C3	-1.04(13)
C1[1]	C1	C6	S2A	-156.1(6)	C6	S2	C9	C8	0.55(14)
C1[1]	C1	C6	C1A	1.2(15)	C6	S2A	C8	C7	11(4)
C1[1]	C1	C6	C7	-158.8(3)	C6	S2A	C8	C9	-1.6(9)
C1	C2	C3	C4	177.12(16)	C6	C1	C2	S1	92.23(16)
C1	C6	C7	C8	-177.65(19)	C6	C1	C2	C1A[1]	-179.2(14)
C1A[1]	C1A	C6	S2	-161(2)	C6	C1	C2	C3	-83.3(2)
C1A[1]	C1A	C6	S2A	39(4)	C6	C7	C8	S2A	-167(5)
C1A[1]	C1A	C6	C1	-1.0(12)	C6	C7	C8	C9	1.8(4)
C1A[1]	C1A	C6	C7	43(4)	C7	C8	C9	S2	-1.4(3)
C1A[1]	C2	C3	C4	-134.9(11)	C8	S2A	C6	S2	2.0(9)
C1A	C6	C7	C8	152(2)	C8	S2A	C6	C1	-176.6(4)
C2	S1	C5	C4	0.48(14)	C8	S2A	C6	C1A	158.3(17)
C2	C1	C6	S2	-153.13(12)	C8	S2A	C6	C7	-11(4)
C2	C1	C6	S2A	25.3(6)	C9	S2	C6	S2A	-1.7(7)
C2	C1	C6	C1A	-177.5(15)	C9	S2	C6	C1	176.67(14)
C2	C1	C6	C7	22.6(3)	C9	S2	C6	C1A	-169.0(9)
C2[1]	C1A	C6	S2	24(2)	C9	S2	C6	C7	0.5(2)

[1]-x,-y,1-z

Table SI8 Hydrogen Atom Coordinates (Å×10[4]) and Isotropic Displacement Parameters (Å[2]×10[3]) for TTE.				
**Atom**	***x***	***y***	***z***	**U(eq)**
H3	-948	3247	3579	18
H4	582	4586	2328	18
H5	3044	3471	2742	18
H7	805	3395	6643	19
H8B	1521	3604	9454	21
H8A	1472	3648	9380	21
H9	1706	1303	10604	21

Table SI9 Atomic Occupancy for TTE.					
**Atom**	***Occupancy***	**Atom**	***Occupancy***	**Atom**	***Occupancy***
S2	0.93	S2A	0.07	C1	0.94
C1A	0.06	C7	0.93	H7	0.93
H8B	0.07	H8A	0.93		

DTE Crystal Data

**Table SI10 Crystal data and structure refinement DTE.**	
Identification code	DTE
Empirical formula	$C_{10}H_8S_2$
Formula weight	192.28
Temperature/K	99.99(10)
Crystal system	monoclinic
Space group	$P2_1/c$
a/Å	5.81321(19)
b/Å	7.5432(3)
c/Å	10.2777(3)
α/°	90
β/°	93.249(3)
γ/°	90
Volume/Å^3	449.95(3)
Z	2
ρ_{calc}g/cm^3	1.419
μ/mm^{-1}	0.526
F(000)	200.0
Crystal size/mm^3	0.25 × 0.15 × 0.1
Radiation	MoKα (λ = 0.71073)
2Θ range for data collection/°	7.944 to 51.994
Index ranges	$-7 \leq h \leq 5, -9 \leq k \leq 8, -12 \leq l \leq 12$
Reflections collected	2262
Independent reflections	848 [R_{int} = 0.0193, R_{sigma} = 0.0232]
Data/restraints/parameters	848/15/57
Completeness to theta = 50°	95.6%
Goodness-of-fit on F^2	1.005
Final R indexes [I>=2σ (I)]	R_1 = 0.0368, wR_2 = 0.0901
Final R indexes [all data]	R_1 = 0.0409, wR_2 = 0.0930
Largest diff. peak/hole / e Å^{-3}	0.45/-0.54

**Table SI11 Fractional Atomic Coordinates (×10⁴) and Equivalent Isotropic Displacement Parameters (Å²×10³) for DTE. U_{eq} is defined as 1/3 of the trace of the orthogonalized U_{IJ} tensor.**				
**Atom**	***x***	***y***	***z***	**U(eq)**
S1	3914.9(12)	6348.1(9)	3605.3(8)	17.5(2)
S1A	827(15)	4672(11)	2227(8)	23(3)
C1	1389(4)	5140(3)	3346(2)	13.3(5)
C1A	2020(30)	5470(20)	3625(15)	9(4)
C2	992(6)	4666(4)	1989(3)	13.1(10)
C2A	4060(30)	6260(30)	3305(15)	14(7)
C3	2864(4)	5345(3)	1275(2)	20.5(5)
C4	4500(4)	6254(3)	2001(2)	19.1(5)
C5	-137(4)	4728(3)	4375(2)	15.6(5)
C5A	930(40)	5360(30)	4880(20)	19(5)

**Table SI12 Anisotropic Displacement Parameters (Å²×10³) for DTE. The Anisotropic displacement factor exponent takes the form: $-2\pi^2[h^2a^{*2}U_{11}+2hka^*b^*U_{12}+…]$.**						
**Atom**	**U_{11}**	**U_{22}**	**U_{33}**	**U_{23}**	**U_{13}**	**U_{12}**
C3	26.2(11)	18.8(10)	16.6(10)	1.6(8)	1.5(8)	5.6(8)
C4	17(1)	15.5(10)	25.2(11)	4.2(7)	3.6(8)	1.1(8)

**Table SI13 Bond Lengths for DTE.**					
**Atom**	**Atom**	**Length/Å**	**Atom**	**Atom**	**Length/Å**
S1	C1	1.736(3)	C1A	C5A	1.472(17)
S1	C4	1.704(2)	C2	C3	1.440(4)
S1A	C1A	1.674(16)	C2A	C4	1.379(14)
S1A	C3	1.658(9)	C3	C4	1.360(3)
C1	C2	1.445(4)	C5	C5[1]	1.349(4)
C1	C5	1.452(3)	C5A	C5A[1]	1.24(5)
C1A	C2A	1.381(14)			
[1]-X,1-Y,1-Z					

Table SI14 Bond Angles for DTE.							
**Atom**	**Atom**	**Atom**	**Angle/°**	**Atom**	**Atom**	**Atom**	**Angle/°**
C4	S1	C1	92.47(11)	C3	C2	C1	108.7(3)
C3	S1A	C1A	96.9(7)	C4	C2A	C1A	116.1(15)
C2	C1	S1	111.4(2)	C4	C3	S1A	109.4(3)
C2	C1	C5	125.4(3)	C4	C3	C2	115.1(2)
C5	C1	S1	123.13(19)	C3	C4	S1	112.29(17)
C2A	C1A	S1A	105.7(12)	C3	C4	C2A	111.8(9)
C2A	C1A	C5A	130.9(17)	C5[1]	C5	C1	125.8(3)
C5A	C1A	S1A	123.4(16)	C5A[1]	C5A	C1A	129(3)
[1]-X,1-Y,1-Z							

Table SI15 Torsion Angles for DTE.									
**A**	**B**	**C**	**D**	**Angle/°**	**A**	**B**	**C**	**D**	**Angle/°**
S1	C1	C2	C3	0.5(3)	C2	C1	C5	C5[1]	-175.0(3)
S1	C1	C5	C5[1]	2.9(4)	C2	C3	C4	S1	0.4(3)
S1A	C1A	C2A	C4	3.0(16)	C2A	C1A	C5A	C5A[1]	180(3)
S1A	C1A	C5A	C5A[1]	2(5)	C3	S1A	C1A	C2A	-0.7(10)
S1A	C3	C4	C2A	3.6(10)	C3	S1A	C1A	C5A	177.2(15)
C1	S1	C4	C3	-0.04(17)	C4	S1	C1	C2	-0.30(19)
C1	C2	C3	C4	-0.6(3)	C4	S1	C1	C5	-178.45(19)
C1A	S1A	C3	C4	-1.7(7)	C5	C1	C2	C3	178.6(2)
C1A	C2A	C4	C3	-4.5(16)	C5A	C1A	C2A	C4	-174.7(18)
[1]-X,1-Y,1-Z									

Table SI16 Hydrogen Atom Coordinates (Å×10^4) and Isotropic Displacement Parameters (Å^2×10^3) for DTE				
**Atom**	***x***	***y***	***z***	**U(eq)**
H2	-289	4015	1629	16
H2A	5104	6765	3946	17
H3B	2885	5130	365	25
H3A	3010(50)	5240(40)	370(30)	25
H4B	5789	6820	1651	23
H4A	5880(50)	6820(40)	1740(30)	23
H5	-1443	4012	4148	19
H5A	1745	5902	5598	23

Table SI17 Atomic Occupancy for DTE.							
**Atom**	***Occupancy***		**Atom**	***Occupancy***		**Atom**	***Occupancy***
S1	0.9		S1A	0.1		C1	0.9
C1A	0.1		C2	0.9		H2	0.9
C2A	0.1		H2A	0.1		H3B	0.1
H3A	0.9		H4B	0.1		H4A	0.9
C5	0.92		H5	0.92		C5A	0.08
H5A	0.08						

sl-TTE Crystal Data

Table SI18 Crystal data and structure refinement for sl-TTE.	
Identification code	sl-TTE
Empirical formula	$C_{18}H_{10}S_4$
Formula weight	354.50
Temperature/K	100.1(5)
Crystal system	monoclinic
Space group	$P2_1/n$
a/Å	10.91901(14)
b/Å	8.76755(11)
c/Å	15.8755(2)
α/°	90
β/°	93.1272(11)
γ/°	90
Volume/Å^3	1517.54(3)
Z	4
ρ_{calc}g/cm^3	1.552
μ/mm^{-1}	5.668
F(000)	728.0
Crystal size/mm^3	0.3 × 0.1 × 0.05
Radiation	CuKα (λ = 1.54184)
2Θ range for data collection/°	9.592 to 133.996
Index ranges	$-8 \leq h \leq 13$, $-10 \leq k \leq 10$, $-18 \leq l \leq 18$
Reflections collected	7920
Independent reflections	2681 [R_{int} = 0.0171, R_{sigma} = 0.0161]
Data/restraints/parameters	2681/0/205
Completeness to theta = 66.5°	99.0%
Goodness-of-fit on F^2	1.002
Final R indexes [I>=2σ (I)]	R_1 = 0.0261, wR_2 = 0.0652
Final R indexes [all data]	R_1 = 0.0274, wR_2 = 0.0661
Largest diff. peak/hole / e Å^{-3}	0.26/-0.28

Table SI19 Fractional Atomic Coordinates (×10⁴) and Equivalent Isotropic Displacement Parameters (Å²×10³) for sl-TTE. U_{eq} is defined as 1/3 of of the trace of the orthogonalised U_{IJ} tensor.				
**Atom**	***x***	***y***	***z***	**U(eq)**
S1	1275.8(3)	3113.8(4)	790.3(2)	18.41(11)
S2	6315.9(3)	4576.6(4)	2582.7(2)	18.34(11)
S3	5254.6(4)	8328.0(4)	1973.5(2)	21.22(11)
S4	2399.4(12)	5729.5(13)	-608.8(5)	20.3(2)
S4A	2242.6(15)	7985.9(13)	740.1(9)	17.7(3)
C1	3257.4(14)	5119.8(17)	1038.1(9)	14.9(3)
C2	4393.9(14)	5454.2(17)	1439.5(9)	15.2(3)
C11	2689.2(14)	3719.3(17)	1223.0(9)	15.5(3)
C12	3193.6(14)	2654.5(17)	1804.8(9)	16.0(3)
C13	2419.5(15)	1345.9(18)	1881(1)	18.7(3)
C14	1375.1(15)	1454.6(18)	1377.4(10)	20.5(3)
C21	4911.9(14)	4383.7(17)	2023.7(9)	15.5(3)
C22	4343.4(14)	3003.5(17)	2223.8(9)	16.2(3)
C23	5057.6(15)	2141.2(18)	2846.5(10)	19.7(3)
C24	6125.6(15)	2847.7(18)	3085.4(10)	22.0(3)
C31	5040.5(13)	6877.0(17)	1242.9(9)	15.6(3)
C32	5471.8(13)	7308.2(17)	464.8(10)	15.9(3)
C33	5993.6(14)	8795.7(19)	500.3(10)	21.5(3)
C34	5932.4(15)	9480.4(19)	1264.3(11)	22.2(3)
C41	2644.1(13)	6195.3(17)	435.4(9)	16.0(3)
C42	2243(6)	7623(6)	593(4)	17.7(3)
C42A	2399(7)	5961(9)	-402(4)	20.3(2)
C43	1720.8(15)	8414.7(19)	-202.0(11)	22.6(3)
C44	1808.2(15)	7393(2)	-831.8(10)	23.5(4)

Table SI20 Anisotropic Displacement Parameters ($Å^2×10^3$) for sl-TTE. The Anisotropic displacement factor exponent takes the form: $-2\pi^2[h^2a^{*2}U_{11}+2hka^*b^*U_{12}+…]$.						
**Atom**	U_{11}	U_{22}	U_{33}	U_{23}	U_{13}	U_{12}
S1	17.51(19)	18.2(2)	19.1(2)	1.02(14)	-2.52(14)	-3.86(14)
S2	18.05(19)	18.4(2)	17.90(19)	0.03(14)	-5.65(14)	0.67(14)
S3	26.6(2)	18.2(2)	18.4(2)	-1.47(14)	-3.26(15)	-4.77(15)
S4	26.7(3)	21.2(5)	12.4(5)	0.3(4)	-4.9(4)	2.1(3)
S4A	22.3(3)	12.4(7)	18.2(7)	0.1(4)	-1.4(4)	3.3(5)
C1	17.5(7)	14.6(7)	12.5(7)	-1.4(6)	-0.2(6)	0.2(6)
C2	18.2(7)	14.5(7)	12.8(7)	-2.2(5)	-0.3(6)	0.5(6)
C11	17.5(7)	16.1(7)	13.0(7)	-2.4(6)	0.2(5)	-0.5(6)
C12	20.1(7)	14.3(7)	13.8(7)	-1.8(6)	1.7(6)	1.0(6)
C13	22.5(8)	16.1(7)	17.5(7)	-0.1(6)	2.3(6)	-0.8(6)
C14	22.3(8)	17.2(8)	22.3(8)	0.3(6)	2.9(6)	-4.9(6)
C21	16.6(7)	16.5(7)	13.1(7)	-3.2(6)	-1.0(5)	1.4(6)
C22	19.8(8)	15.0(7)	13.8(7)	-1.7(6)	0.4(6)	1.7(6)
C23	25.0(8)	16.2(7)	17.5(7)	0.7(6)	-1.2(6)	1.8(6)
C24	26.8(8)	19.5(8)	18.9(8)	1.8(6)	-4.9(6)	4.8(6)
C31	14.8(7)	15.0(7)	16.5(7)	-1.2(6)	-4.4(6)	0.9(6)
C32	12.8(7)	13.5(7)	21.2(7)	-0.2(6)	0.3(6)	0.8(6)
C33	16.4(7)	23.6(8)	24.2(8)	7.2(6)	-1.2(6)	-1.4(6)
C34	19.0(8)	17.7(8)	29.3(9)	2.3(6)	-6.0(6)	-3.3(6)
C41	14.9(7)	16.9(7)	15.8(7)	1.1(6)	-1.9(6)	-3.3(6)
C42	22.3(3)	12.4(7)	18.2(7)	0.1(4)	-1.4(4)	3.3(5)
C42A	26.7(3)	21.2(5)	12.4(5)	0.3(4)	-4.9(4)	2.1(3)
C43	17.9(8)	18.6(8)	30.9(9)	2.4(7)	-2.4(6)	-1.3(6)
C44	21.1(8)	28.7(9)	20.1(8)	3.2(7)	-4.4(6)	-4.3(7)

Table SI21 Bond Lengths for sl-TTE.

Atom	Atom	Length/Å		Atom	Atom	Length/Å
S1	C11	1.7378(15)		C11	C12	1.405(2)
S1	C14	1.7280(16)		C12	C13	1.434(2)
S2	C21	1.7372(15)		C12	C22	1.422(2)
S2	C24	1.7308(17)		C13	C14	1.360(2)
S3	C31	1.7288(15)		C21	C22	1.404(2)
S3	C34	1.7108(17)		C22	C23	1.440(2)
S4	C41	1.7140(16)		C23	C24	1.356(2)
S4	C44	1.626(2)		C31	C32	1.398(2)
S4A	C41	1.7069(17)		C32	C33	1.423(2)
S4A	C43	1.615(2)		C33	C34	1.358(2)
C1	C2	1.395(2)		C41	C42	1.354(6)
C1	C11	1.414(2)		C41	C42A	1.357(7)
C1	C41	1.478(2)		C42	C43	1.524(7)
C2	C21	1.416(2)		C42A	C44	1.553(8)
C2	C31	1.475(2)		C43	C44	1.350(3)

Table SI22 Bond Angles for sl-TTE.

Atom	Atom	Atom	Angle/°		Atom	Atom	Atom	Angle/°
C14	S1	C11	90.90(7)		C21	C22	C12	118.01(14)
C24	S2	C21	91.46(8)		C21	C22	C23	112.23(14)
C34	S3	C31	92.33(8)		C24	C23	C22	112.18(14)
C44	S4	C41	92.06(9)		C23	C24	S2	113.20(12)
C43	S4A	C41	91.99(9)		C2	C31	S3	121.95(11)
C2	C1	C11	118.51(14)		C32	C31	S3	110.73(11)
C2	C1	C41	121.48(13)		C32	C31	C2	127.15(13)
C11	C1	C41	120.01(13)		C31	C32	C33	111.49(14)
C1	C2	C21	118.27(14)		C34	C33	C32	113.66(14)
C1	C2	C31	120.35(13)		C33	C34	S3	111.76(12)
C21	C2	C31	121.37(13)		C1	C41	S4	121.03(12)
C1	C11	S1	125.00(12)		C1	C41	S4A	121.18(12)
C12	C11	S1	111.40(11)		C42	C41	S4	111.4(3)
C12	C11	C1	123.58(14)		C42	C41	C1	127.6(3)
C11	C12	C13	112.05(14)		C42A	C41	S4A	112.1(4)
C11	C12	C22	117.99(14)		C42A	C41	C1	126.6(4)
C22	C12	C13	129.96(14)		C41	C42	C43	112.2(4)
C14	C13	C12	111.94(14)		C41	C42A	C44	111.3(5)
C13	C14	S1	113.70(12)		C44	C43	S4A	119.72(14)
C2	C21	S2	125.46(12)		C44	C43	C42	105.9(2)
C22	C21	S2	110.91(11)		C43	C44	S4	118.50(13)
C22	C21	C2	123.63(14)		C43	C44	C42A	104.8(3)
C12	C22	C23	129.77(15)					

**Table SI23 Torsion Angles for sl-TTE**										
**A**	**B**	**C**	**D**	**Angle/°**		**A**	**B**	**C**	**D**	**Angle/°**
S1	C11	C12	C13	0.67(16)		C12	C13	C14	S1	0.26(18)
S1	C11	C12	C22	-178.95(11)		C12	C22	C23	C24	178.81(15)
S2	C21	C22	C12	-178.68(11)		C13	C12	C22	C21	179.76(15)
S2	C21	C22	C23	1.24(16)		C13	C12	C22	C23	-0.1(3)
S3	C31	C32	C33	1.62(16)		C14	S1	C11	C1	-178.81(14)
S4	C41	C42	C43	-1.4(5)		C14	S1	C11	C12	-0.45(12)
S4A	C41	C42A	C44	-0.7(6)		C21	S2	C24	C23	0.21(13)
S4A	C43	C44	C42A	2.3(4)		C21	C2	C31	S3	-68.83(18)
C1	C2	C21	S2	179.55(11)		C21	C2	C31	C32	116.35(17)
C1	C2	C21	C22	-0.2(2)		C21	C22	C23	C24	-1.1(2)
C1	C2	C31	S3	112.33(14)		C22	C12	C13	C14	178.96(15)
C1	C2	C31	C32	-62.5(2)		C22	C23	C24	S2	0.46(18)
C1	C11	C12	C13	179.07(14)		C24	S2	C21	C2	179.40(14)
C1	C11	C12	C22	-0.6(2)		C24	S2	C21	C22	-0.84(12)
C1	C41	C42	C43	176.8(2)		C31	S3	C34	C33	0.13(13)
C1	C41	C42A	C44	-176.9(2)		C31	C2	C21	S2	0.7(2)
C2	C1	C11	S1	179.64(11)		C31	C2	C21	C22	-179.04(14)
C2	C1	C11	C12	1.5(2)		C31	C32	C33	C34	-1.58(19)
C2	C1	C41	S4	115.15(15)		C32	C33	C34	S3	0.80(18)
C2	C1	C41	S4A	-60.68(19)		C34	S3	C31	C2	-176.61(13)
C2	C1	C41	C42	-62.8(4)		C34	S3	C31	C32	-1.02(12)
C2	C1	C41	C42A	115.2(5)		C41	S4	C44	C43	-0.42(16)
C2	C21	C22	C12	1.1(2)		C41	S4A	C43	C44	-2.43(17)
C2	C21	C22	C23	-178.99(14)		C41	C1	C2	C21	178.57(13)
C2	C31	C32	C33	176.92(14)		C41	C1	C2	C31	-2.6(2)
C11	S1	C14	C13	0.11(13)		C41	C1	C11	S1	0.0(2)
C11	C1	C2	C21	-1.1(2)		C41	C1	C11	C12	-178.17(14)
C11	C1	C2	C31	177.81(13)		C41	C42	C43	C44	1.0(5)
C11	C1	C41	S4	-65.22(18)		C41	C42A	C44	C43	-0.9(6)
C11	C1	C41	S4A	118.95(15)		C42	C43	C44	S4	-0.3(3)
C11	C1	C41	C42	116.8(4)		C43	S4A	C41	C1	178.06(13)
C11	C1	C41	C42A	-65.2(5)		C43	S4A	C41	C42A	1.7(4)
C11	C12	C13	C14	-0.60(19)		C44	S4	C41	C1	-177.22(13)
C11	C12	C22	C21	-0.7(2)		C44	S4	C41	C42	1.0(3)
C11	C12	C22	C23	179.39(15)						

Table SI24 Hydrogen Atom Coordinates (Å×10⁴) and Isotropic Displacement Parameters (Å²×10³) for sl-TTE.				
**Atom**	***x***	***y***	***z***	**U(eq)**
H13	2613	504	2240	22
H14	758	690	1351	25
H23	4809	1188	3065	24
H24	6710	2435	3488	26
H32	5423	6686	-26	19
H33	6351	9266	34	26
H34	6230	10478	1390	27
H42	2283	8079	1137	21
H42A	2567	5039	-688	24
H43A	1354	9382	-312	27
H43	1400	9420	-245	27
H44	1531	7640	-1393	28
H44A	1563	7522	-1411	28

Table SI25 Atomic Occupancy for sl-TTE.					
**Atom**	***Occupancy***	**Atom**	***Occupancy***	**Atom**	***Occupancy***
S4	0.55	S4A	0.45	C42	0.55
H42	0.55	C42A	0.45	H42A	0.45
H43A	0.45	H43	0.55	H44	0.55
H44A	0.45				

Materials and general information

Tetrathienylethene TTE has been previously synthesized by McMurry coupling at *Universita' degli Studi di Milano*, in the laboratory and under the supervision of Professor Emanuela Licandro, and used as such for the experiments, subject of this work, after checking its purity by HPLC.

THF was distilled under normal pressure from sodium benzophenone ketyl, under nitrogen immediately prior to use. The reaction outcome was monitored by TLC silica gel plates, 60, F254. The purity of the investigated molecules was checked both ^{1}H-NMR and HPLC. ^{1}H-NMR spectra were performed by using Acetone-d_6 as solvent, tetramethylsilane (TMS δ=0 ppm) as the internal reference and by adding some drops of CS_2 in sl-TTE and fl-TTE case to promote the solubility of the molecule in the deuterated solvent. HPLC chromatograms were recorded in reverse phase, isocratic conditions, by using as eluent the mixture CH_3CN:H_2O 6:4 or 9:1 (1 mL / min).

Instruments used for the characterization of sl-TTE and fl-TTE and for the photoluminescence studies.

^{1}H NMR spectra were recorded on a Bruker ARX 400 NMR spectrometer. Mass spectra were recorded on a GCT premier CAB048 mass spectrophotometer operating in a MALDI-TOF mode. UV-vis absorption spectra were recorded on LIBRA BIOCHROME spectrophotometer. Photoluminescence spectra were recorded on a Perkin-Elmer LS 55 spectrofluorometer. The solid state fluorescence quantum yield were measured by the absolute PL quantum yield spectrometer C11347 of Hamamatsu with a calibrated integrating sphere. The lifetime were determined by the compact fluorescence lifetime spectrometer C11367 of Hamamatsu. HPLC chromatograms were performed on an Agilent 1260 Infinity equipped with a PDA detector and the reverse phase column ZORBAX SB-C_{18} 4.6 X 150 mm, 5 μm particle size.

Experimental Procedures

Synthesis of 4,5-bis(thiophene-2-yl)thieno[3,2-e]benzo[b]thiophene (semi-locked TTE; sl-TTE) and tetrathieno[2,3-a:3',2'-c:2'',3''-f:3''',2'''-h]-naphthalene (fully-locked TTE; fl-TTE)[1]

In a typical run for photochemical reactions, 80 mg (0.217 mmol, 1 eq) of tetrakis(2-thienyl)ethene (TTE) were dissolved in 200 mL of toluene (solution color: yellow) and the solution was degassed under N_2 atmosphere for 30 min (Scheme 1). After this time, 116 mg (0.457 mmol, 2 eq) of I_2 were added to the solution (which turned its color from yellow to red) and it was degassed again for other 30 min. Then 50 mL (714 mmol, d=0.83 g mL-1) of 2-methyl oxirane (propylene oxide) were added

in the reaction mixture and the solution was degassed for 30 min more. The resulting mixture was irradiated with UV-light from a 500 W high pressure Hg vapor lamp placed in the immersion quartz well under N2 flow. The reaction was monitored by TLC (eluent: hexane/DCM 8/2) and it was carried on until the consumption of the starting material and the appearance of the final product. The overall reaction time was 20 min. The reaction product precipitated during the photolysis due to its insolubility in the reaction solvent. The crude was washed with DCM and ethanol to remove the alcohol (2-iodoethan-1-ol) byproduct of this reaction, which is soluble in this medium, contrary to the reaction products which are insoluble both in DCM and in ethanol. By washing the residual crude with hexane, it was possible to isolate the intermediate product (4,5-bis(thiophene-2-yl)thieno[3,2-e]benzo[b]thiophene (semi-locked TTE; sl-TTE). By recrystallizing once, the remaining crude from o-xylene the final desired product tetrathieno[2,3-a:3',2'-c:2'',3''-f:3'',2''-h]-naphthalene (fully-locked TTE; fl-TTE) (50 mg 64% yield) was obtained as a micro-crystalline powder. (fl-TTE) 1H-NMR (400 MHz, some drops of CS2 + Acetone d6): δ, ppm = 8.09 (d, 1H, J = 4.8 Hz), 7.94 (d, 1H, J = 5.2 Hz), UV/Vis (THF), λ/nm: 338; ε, M-1 cm-1: 18.6 E4; optical energy gap: 3.67 eV; MS: 352.9 [M+]; HPLC (reverse phase, analytical column SB C18, eluent: ACN/H2O 9/1) retention time: 7.12 min.

(sl-TTE) 1H-NMR (400 MHz, some drops of CS_2 + Acetone d6): δ, ppm = 7.99 (d, 1H, J = 4.8 Hz), 7.86 (d, 1H, J = 4.8 Hz), 7.54 (d, 1H, J = 4.8 Hz), 7.25 (bs, 1H), 7.11 (bs, 1H), UV/Vis (THF), λ/nm: 320; ε, M-1 cm-1: 7.73 E^3; optical energy gap: 3.88 eV; MS: 354.9 [M+].

Single crystals of $C_{18}H_{12}S_4$ (TTE), $C_{18}H_8S_2$ (DTE) and $C_{18}H_{10}S_4$, sl-TTE.

TTE and DTE crystals were grown by dissolving the sample in dichloromethane in which they have a very good solubility. Then a co-layer of another solvent (hexane in the case of TTE, methanol in case of DTE) was added very slowly in order to create an interface between the two solvents. The slow diffusion of the co-solvent in which the molecules are not soluble made possible the single crystal growth, which was obtained after one week.

sl-TTE was grown by evaporation from THF.

For the data collection of TTE, DTE, and sl-TTE suitable crystals were selected and mounted on a SuperNova, Dual, Cu at zero, Atlas diffractometer. The crystal was kept at 100.0K during data collection. Using Olex2[2] the structure was solved with the ShelXS[3] structure solution program using Direct Methods and refined with the ShelXL[4] refinement package using Least Squares minimization.

Crystal data for $C_{18}H_{12}S_4$, TTE, (MM =356.52 g/mol): monoclinic, space group P21/n (no. 14), a= 9.3160(2) Å, b = 9.10329(18) Å, c = 9.6346(2) Å, β = 110.853(3)° , V = 763.55(3) Å3, Z = 2, T = 100.0(4) K, μ(CuKα) = 5.633 mm-1, Dcalc= 1.551 g/cm3, 3875 reflections measured (11.34° ≤ 2Θ

≤ 133.94°), 1353 unique (Rint = 0.0145, Rsigma = 0.0151) which were used in all calculations. The final R1 was 0.0250 (>2σ (I)) and wR2 was 0.0648 (all data).

Crystal data for $C_{10}H_8S_2$, DTE (M =192.28 g/mol): monoclinic, space group P21/c (no. 14), a = 5.81321(19) Å, b = 7.5432(3) Å, c = 10.2777(3) Å, β = 93.249(3)°, V = 449.95(3) Å3, Z = 2, T = 99.99(10) K, μ(MoKα) = 0.526 mm-1, Dcalc = 1.419 g/cm3, 2262 reflections measured (7.944° ≤ 2Θ ≤ 51.994°), 848 unique (Rint = 0.0193, Rsigma = 0.0232) which were used in all calculations. The final R1 was 0.0368 (I > 2σ (I)) and wR2 was 0.0930 (all data).

Crystal Data for $C_{18}H_{10}S_4$, sl-TTE (M =354.50 g/mol): monoclinic, space group P21/n (no. 14), a = 10.91901(14) Å, b = 8.76755(11) Å, c = 15.8755(2) Å, β = 93.1272(11)°, V = 1517.54(3) Å3, Z = 4, T = 100.1(5) K, μ(CuKα) = 5.668 mm-1, Dcalc = 1.552 g/cm3, 7920 reflections measured (9.592° ≤ 2Θ ≤ 133.996°), 2681 unique (Rint = 0.0171, Rsigma = 0.0161) which were used in all calculations. The final R1was 0.0261 (I > 2σ(I)) and wR2 was 0.0661 (all data).

Refinement model description

TTE

There is a disorder along the rotation axis of the thiophene group S(1), C2(2), C(3), C(4) and C(5), in which the positions of S(2) and C(7) are interconvertible. The occupancies of S(2) and C(7) were found to be 0.93 and 0.07 respectively, and the counterpart of S(2), named as S(2A) was refined as 0.07 for a converged refinement. On the other hand, the counter part of C(7) was not located, a model was tried but the refinement is never stable, probably due to the too small portion of disorder part of C(7) which is shielded by the nearby S(2) atom with large electron density.

Another disorder found at C(1), its counterpart was named as C(1A), with occupancies 0.94 and 0.06 respectively.

The model indicated that there is another position of the double bond bridge which is perpendicular to the major one.

DTE

There is a flipping disorder on the plane of S(1) C(2), C(3), C(4) and C(5), in which the positions of S(2) and C(2) are mostly interconvertible. The occupancies of S(2), C(1) and C(2) were found to be 0.90 and 0.10 respectively. The counterpart of S(2), C(1) and C(2), named S(2A), C(1A) and C(2A) respectively, were refined as 0.10 for a converged refinement. The model indicated that there is another position of the double bond bridge C(5) = C(5), which is perpendicular to the major one with occupancy 0.92 for the major part and 0.08 for the minor.

sl-TTE

There is a disorder along the rotation axis of the thiophene group S(4), C(41), C(42), C(43) and C(44) which parallel to the bond C(1) and C(41), in which the positions of S(4) and C(42) are interconvertible.

The occupancies of part1 (named S(4) and C(42)) and part2 (named S(4A) and C(42A)) were found to be 0.55 and 0.45 respectively.

1. E. Fischer, J. Larsen, J. B. Christensen, M. Fourmigué, H. G. Madsen and N. Harrit, *J. Org. Chem.*, 1996, **61**, 6997-7005.
2. O. V. Dolomanov, L. J. Bourhis, R. J. Gildea, J. A. K. Howard and H. Puschmann, *J. Appl. Crystallogr.*, 2009, **42**, 339-341.
3. G. Sheldrick, *Acta Crystallographica Section A*, 2008, **64**, 112-122.
4. G. Sheldrick, *Acta Crystallographica Section C*, 2015, **71**, 3-8.
